# How a 10-*epi*-Cubebol Synthase Avoids Premature Reaction Quenching to Form a Tricyclic Product at High Purity

**DOI:** 10.1021/acscatal.2c03155

**Published:** 2022-09-21

**Authors:** Joshua N. Whitehead, Nicole G. H. Leferink, Gajendar Komati Reddy, Colin W. Levy, Sam Hay, Eriko Takano, Nigel S. Scrutton

**Affiliations:** † Manchester Institute of Biotechnology, Department of Chemistry, The University of Manchester, Manchester M1 7DN, U.K.; ‡ Future Biomanufacturing Research Hub (FBRH), Manchester Institute of Biotechnology, Department of Chemistry, The University of Manchester, Manchester M1 7DN, U.K.

**Keywords:** terpene synthase, protein engineering, protein crystallography, carbocation stabilization, sesquiterpenoids, mechanism

## Abstract

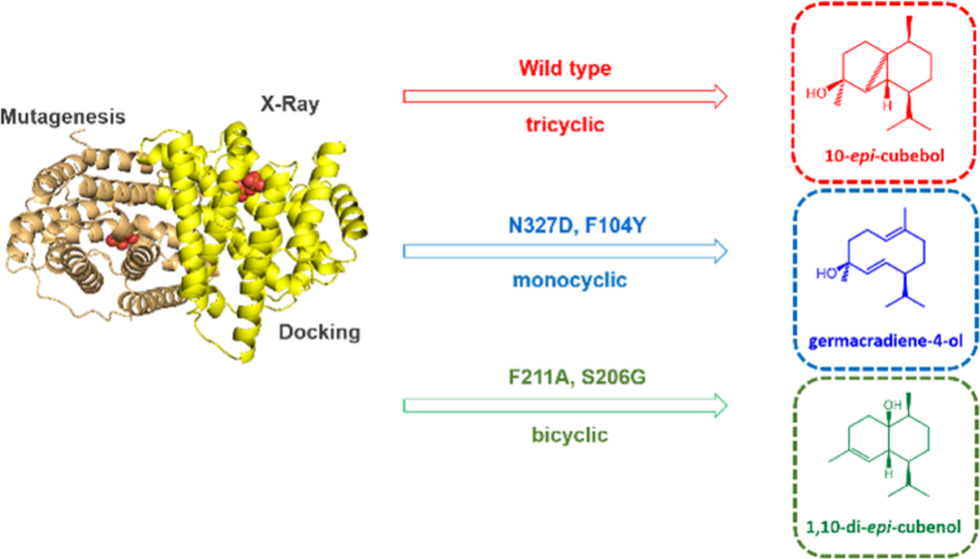

Terpenes are the largest class of natural products and are attractive targets in the fuel, fragrance, pharmaceutical, and flavor industries. Harvesting terpenes from natural sources is environmentally intensive and often gives low yields and purities, requiring further downstream processing. Engineered terpene synthases (TSs) offer a solution to these problems, but the low sequence identity and high promiscuity among TSs are major challenges for targeted engineering. Rational design of TSs requires identification of key structural and chemical motifs that steer product outcomes. Producing the sesquiterpenoid 10-*epi*-cubebol from farnesyl pyrophosphate (FPP) requires many steps and some of Nature’s most difficult chemistry. 10-*epi*-Cubebol synthase from *Sorangium cellulosum* (ScCubS) guides a highly reactive carbocationic substrate through this pathway, preventing early quenching and ensuring correct stereochemistry at every stage. The cyclizations carried out by ScCubS potentially represent significant evolutionary expansions in the chemical space accessible by TSs. Here, we present the high-resolution crystal structure of ScCubS in complex with both a trinuclear magnesium cluster and pyrophosphate. Computational modeling, experiment, and bioinformatic analysis identified residues important in steering the reaction chemistry. We show that S206 is crucial in 10-*epi*-cubebol synthesis by enlisting the nearby F211 to shape the active site contour and prevent the formation of early escape cadalane products. We also show that N327 and F104 control the distribution between several early-stage cations and whether the final product is derived from the germacrane, cadalane, or cubebane hydrocarbon scaffold. Using these insights, we reengineered ScCubS so that its main product was germacradien-4-ol, which derives from the germacrane, rather than the cubebane, scaffold. Our work emphasizes that mechanistic understanding of cation stabilization in TSs can be used to guide catalytic outcomes.

## Introduction

Terpenoids, or isoprenoids, are the largest class of natural products, with over 80,000 compounds known to date.^1,2^ Many of these compounds are industrially valuable, for example, as flavors and fragrances, and as precursors for pharmaceuticals, bioplastics, and next-generation jet fuels.^3,4^ Terpene synthases (TSs) are the enzymes responsible for the considerable structural diversity found in terpenoids. They achieve this by catalyzing the conversion of a single linear, isoprenoid pyrophosphate precursor into a highly reactive, cationic hydrocarbon skeleton. After initial pyrophosphate abstraction, the cationic intermediate can undergo multiple changes including hydride shifts, intramolecular cyclization, and Wagner–Meerwein rearrangements, before being terminated by nucleophilic quenching or hydrogen abstraction.^5^ Unusually for enzymes, which typically act by rate enhancement, the challenge for TSs is to “control” the reaction chemistry of these cationic intermediates. For this reason, TSs often give complex product mixtures, and the major product of even high-fidelity TSs can be changed with small modifications to the active site architecture.^6−8^

TSs typically belong to one of two main classes (class I and class II), each with its own substrate ionization mechanism and evolutionarily distinct α-helical fold.^9−11^ In class I TSs, the isoprenoid substrate is activated by coordination of the pyrophosphate moiety (PPi) to a trinuclear Mg^2+^ cluster, which is itself bound by two highly conserved motifs: the aspartate-rich DDxxD/E motif and the NSE/DTE triad.^12−15^ The considerable chemical diversity achieved by TSs stems from a highly branched reaction cascade^16^ where, after initial ionization, the enzyme active site acts mainly as a hydrophobic mold for directing the evolving substrate to the final product(s).^12^ Because of this, and the highly subtle chemical effects employed by TSs to guide product formation, sequence identity across TSs is generally low; classification to date has depended more on phylogeny than product profile, especially for plant TSs. This makes product prediction and rational design of TSs extremely challenging, undermining the industrialization of microbially produced terpenes. Instead, the small but growing number of class I and class II TS crystal structures^17−20^ must be interrogated closely, and a multidisciplinary approach^21^ used to deduce how exactly members of this enzyme class turn their isoprenoid precursors into the many thousands of terpenoids observed in nature.

**Figure 1:**
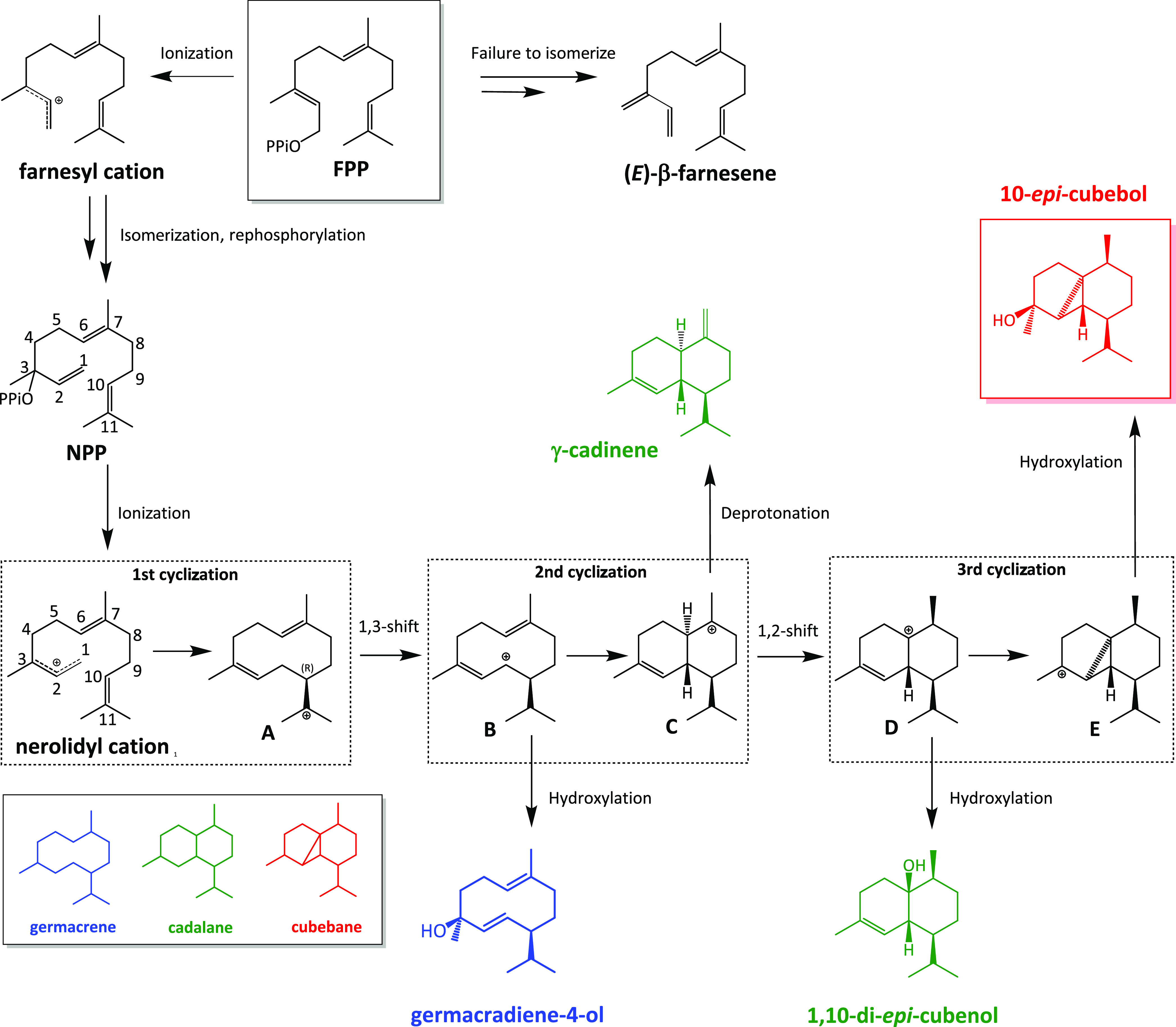
Proposed mechanism for the formation of 10-*epi*-cubebol and key side products from FPP by ScCubS. Relative distribution between cations A–E determines the outcome of the carbon scaffold. Germacranes (blue) are derived from cations A and B; cadalanes (green) from cations C and D; cubebanes (red) from cation E. The three cyclization steps are shown in dashed boxes. The hydrocarbon skeletons of germacranes, cadalanes, and cubebanes are shown in the bottom left box.

The soil-dwelling, Gram-negative myxobacterium *Sorangium cellulosum* (So ce56) produces many different volatile sesquiterpenoids and has one of the largest bacterial genomes, encoding three class I and one class II TS genes.^22^ Sce6369 has been defined as a class I 10-*epi*-cubebol synthase (ScCubS), and is responsible for most of the sesquiterpenoids observed in the volatile extract of *S. cellulosum* So ce56.^23^ ScCubS is capable of making over 20 different sesquiterpenoids when expressed in a heterologous *Escherichia coli* host,^23,24^ the majority of which are derived from the germacrane, cubebane, and cadalane hydrocarbon skeletons (Figure 1). Wild-type ScCubS (WT) generates 10-*epi-*cubebol at high purity, representing around 90% of all of the sesquiterpenoids produced. Although promiscuous in terms of the number of different products made, ScCubS clearly exerts high levels of control to achieve this main product purity. The other sesquiterpenoids detected were cis-muurola-3,5-diene, (*E*)-β-farnesene, germacrene D, cubebol, germacradien-4-ol, γ-cadinene, and five unidentified sesquiterpenes, believed to be isomers of cubebol and *epi*-cubebol. Recent studies have shown that ScCubS, in addition to making sesquiterpenoids, is also capable of producing the monoterpenoids β-pinene, β-mycrene, β-cis- and trans-ocimene, and linalool when expressed in an *E. coli* strain containing a heterologous isoprenoid production pathway, and a heterologous geranyl-diphosphate (GPP) synthase.^24^

10-*epi*-Cubebol is a very complex molecule, and ScCubS has somehow evolved to prevent quenching during the three cyclizations and multiple hydride shifts required to achieve it (Figure 1, full description in the Supporting Information).

One can imagine TSs evolving the ability to carry out these steps sequentially. Coordination of the PPi moiety is the most fundamental step, proven by the conserved localization of the metal-binding motifs. Ionized FPP can be converted to farnesol or farnesene, depending on whether the reaction is quenched by water attack or deprotonation. Isomerization was probably the next step to evolve and depends on the similarly conserved, previously described “sensor-linker-effector” triad.^25^ Isomerization of the C2,3 bond of FPP significantly increases the opportunities for cyclization, bringing the positive charge close to the double bonds along the substrate chain. Cyclization provides a considerable expansion in the chemical space accessible by TSs. The nerolidyl cation contains two double bonds, either of which can attack the positive charge to give 1,6 and 1,10 ring closure, respectively. Similarly, one intramolecular cyclization can be followed by another. Each cyclization generates a novel hydrocarbon scaffold, each more complex than the last, but at all times, the carbocationic intermediate is susceptible to quenching. TSs like ScCubS must have evolved ways to protect the transient positive charge as it moves around the substrate, and therefore represent useful case studies for enzyme engineering.

To gain insight into this, we report the crystal structure of ScCubS in complex with a trinuclear magnesium cluster and pyrophosphate. Based on the crystal structure and our computational modeling, we targeted several active site residues believed to be involved in guiding the reactive carbocation intermediates. This targeted library of mutants was tested for altered product profiles in our previously established “plug-and-play” *in vivo* terpenoid production platform.^26^ This more focused approach is in contrast to less targeted high-throughput methods and negates the need for extensive mutant libraries and multiple rounds of enzyme variant screening. The insights gained here should help with the future rational design of terpene synthases.

## Results and Discussion

### Crystal Structures and Modeling

**Figure 2:**
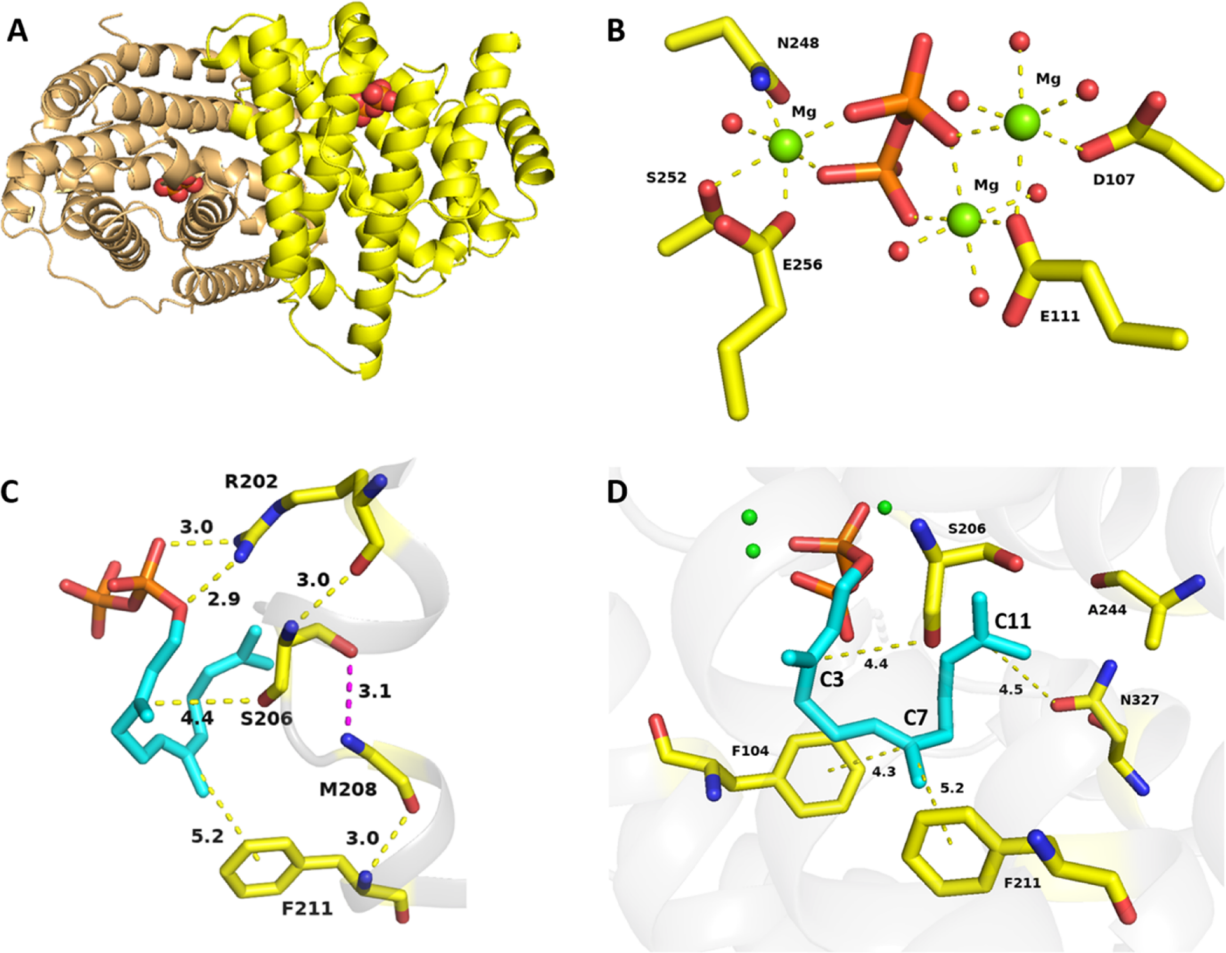
Crystal structure and docking models of ScCubS. (A) ScCubS-Mg_3_^2+^-PPi crystal homodimer (PPi = red). (B) Detail from the ScCubS-Mg_3_^2+^-PPi complex, showing the octahedral coordination of the trinuclear Mg^2+^ cluster (green) to PPi (red) and part of the metal-binding motif (yellow). (C) ScCubS-Mg_3_^2+^-FPP model. The PPi sensor R202 is hydrogen-bonded to the effector S206 (the linker T205 is omitted for clarity). When FPP (blue) is sensed, the G-helix (gray ribbon) moves toward the substrate, triggering ionization via the S206 carbonyl.^25^ S206 occupies the flexible helix-break loop. The side chain of S206 is at the center of a hydrogen-bonding network that connects R202 to F211. The key bond to M208 is shown in magenta. (D) ScCubS-Mg_3_^2+^-FPP docking model, showing the residues chosen for mutation and how they interact with FPP.

The crystal structure of ScCubS in complex with the trinuclear Mg^2+^ cluster and PPi was determined at 1.80 Å resolution (Figure 2A,B). ScCubS crystallizes as a homodimer and exhibits the class I TS structural features, comprising a bundle of 17 α-helices and a central hydrophobic cavity, showing structural similarity to other solved bacterial sesquiterpene synthases (Figure S1). The atomic coordinates and structure factors have been deposited in the Protein Data Bank with accession code 7ZRN, and a detailed description of the crystal structure is given in the Supporting Information.

Docking of FPP into the ScCubS-Mg_3_^2+^-PPi complex (PPi removed) and sequence alignment with previously characterized TSs suggested several residues that could provide the highly tuned control observed in ScCubS (Figure 2D), namely, F104, S206, F211, A244, and N327. The A244 variants were characterized but were unrevealing and are not discussed here (titers for all products and variants are given in Table S4). In TSs, the ligand often binds in a product-like or intermediate-like conformation, with the active site contour dictating to a large extent the shape of the final product. The similarity of the docking conformation with the early intermediates and the results of the mutagenesis experiments give confidence to the computationally derived binding pose (Figure S3).

### N327 and F104 Unlock the Second Intramolecular Cyclization

Isomerization of the farnesyl cation to the nerolidyl cation permits the first intramolecular cyclization and the formation of cation A. Subsequent cyclizations generate novel hydrocarbon scaffolds (Figures S7–S43), but these steps must outcompete the quenching of the substrate. The polar side chain of N327 points toward C11 of FPP (Figure 2D), the position at which the first cyclic intermediate contains a positive charge (Figure 1). It also binds a water molecule close to this position. This suggested a role for N327 in incorporating water into the final product and/or stabilizing the first cyclic intermediate.

**Figure 3:**
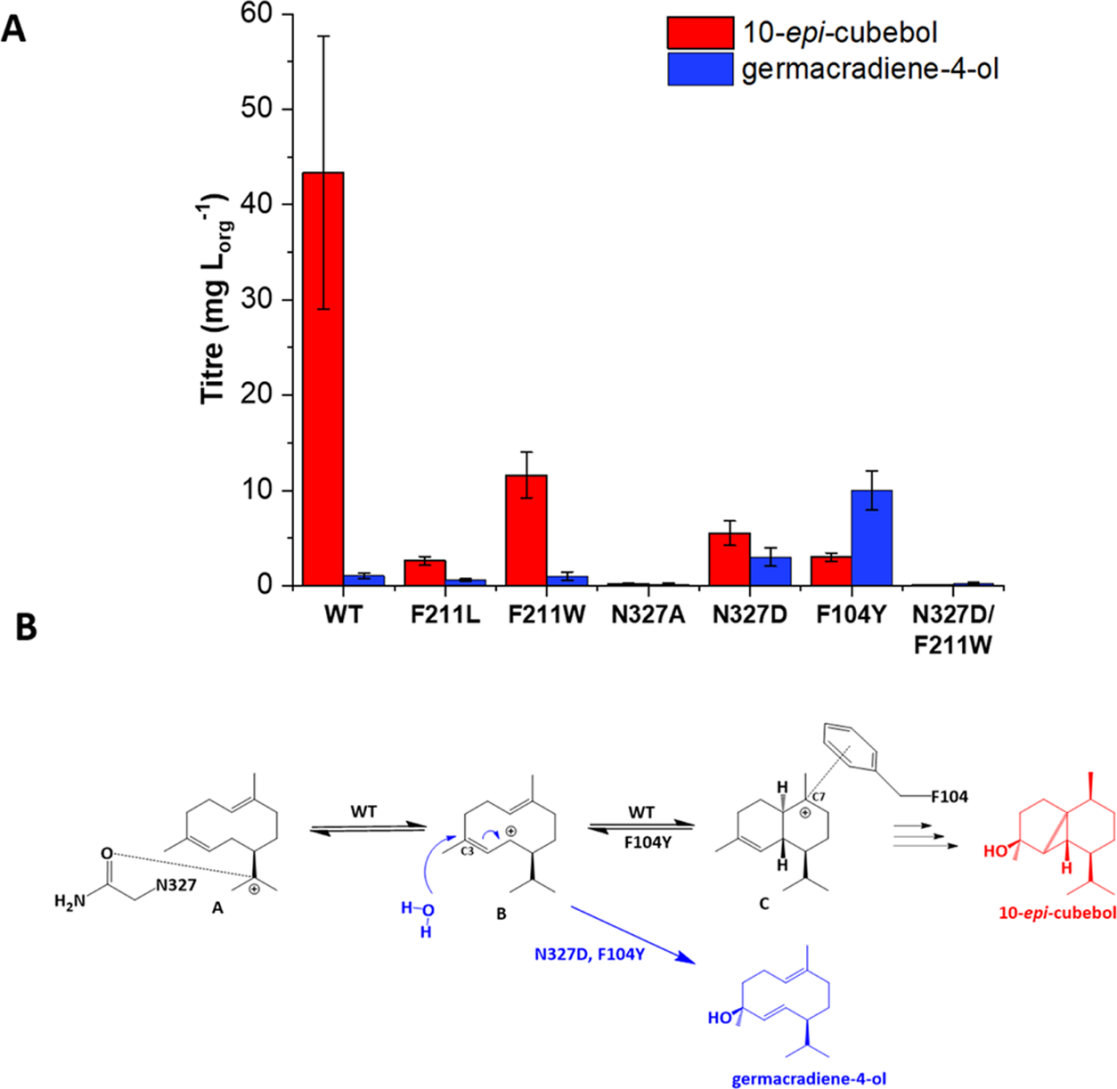
Competing pathways for cation B. (A) Titers of 10-*epi*-cubebol and Gd4ol in several variants. Average titers are calculated from a minimum of two to six replicates and are given in mg/L of organic overlay. F104Y is effectively a germacradien-4-ol synthase. (B) Intramolecular cyclization of cation B gives cation C and continues the cascade. Hydroxylation of cation B at C3 gives Gd4ol. In the WT, N327 and F104 work in tandem to achieve cation C. N327D and F104Y make increased amounts of Gd4ol by stabilizing cation B and destabilizing cation C, respectively.

In our nonpolar variant N327A, we detected only two products: the main product 10-*epi*-cubebol (56% of total products) and germacradien-4-ol (Gd4ol, 44%), although the total sesquiterpene titer was a meager ∼0.4 mg/L organic overlay (mg/L_org_), compared to ∼50.0 mg/L_org_ for WT ScCubS (Figure 3A), suggesting N327 was more fundamentally important to the overall mechanism. N327 corresponds to N305 in a previously characterized bacterial 1,8-cineole synthase (bCinS), where mutation to a nonpolar residue similarly destroyed the ability of the enzyme to make its major product.^27^

In N327D, the same change in product profile was observed at healthier titers. For this variant, we detected the main product 10-*epi*-cubebol at 55% and Gd4ol at 30% of the total sesquiterpene titer, which was ∼10.0 mg/L_org_. We propose that Gd4ol is achieved by the premature quenching of cation B, as shown in Figure 3B, consistent with a previously described high-fidelity germacradien-4-ol synthase.^28^

It is notable that this premature quenching takes place at C3, the same position at which the main product 10-*epi-*cubebol is hydroxylated. A positive charge accumulates at C11 after the first cyclization, after which a 1,3 hydride shift converts cation A to cation B (Figure 1). At this stage, the positive charge is distributed across an allylic system, with S206 and N327 well placed to offer additional stabilization via their main-chain and side-chain carbonyls, respectively. N327A lacks this side-chain group, resulting in low activity. The side chain of N327D, however, with its explicit negative charge, presumably interacts more strongly with the C3/C11 positive charge systems than the WT asparagine, keeping the distribution more in favor of cations A and B, at the expense of cation C. The subsequently increased lifetime of cation B, and perhaps a more polarized coordinated water molecule, allows quenching to occur, delivering Gd4ol and terminating the reaction. In the WT, as we will show, the positive charge is encouraged to move around the substrate by judiciously placed amino acid residues. Finally, in the penultimate step of 10-*epi*-cubebol synthesis, the positive charge accumulates again on C3. However, this time no other rearrangements are possible, and hydroxylation of this position is highly favored. If this is the case, the choice of the moderately polar asparagine (vs a negatively charged or nonpolar residue) is highly specific, and is crucial to 10-*epi*-cubebol synthesis by providing sufficient stability for the conversion of the nerolidyl cation to cation A, but not preventing the subsequent conversion of cation B to C, the second intramolecular cyclization. The effector residue (discussed below) is another such example of this feature in TSs, in which a carbonyl stabilizes positive charge without forming explicit covalent or ionic bonds. The recurrence of this moderately polar motif in an otherwise hydrophobic active site is typical of TSs.^29^

To test these insights, we tried to rationally engineer ScCubS to flip its product specificity towards Gd4ol. Gd4ol was achieved at the highest proportions in our N327D, F211L, and F211W variants (F211 is discussed below). It is worth noting that F211 corresponds to an isoleucine in a previously reported germacradien-4-ol synthase (GdolS), although mutating this isoleucine had almost no effect on product profile,^28^ illustrating perfectly the difficulty in engineering TSs from sequence information alone.

With this in mind, we designed two double-point variants, N327D/F211L and N327D/F211W, but these multiple changes to the active site were tolerated poorly by the enzyme. F211L/N327D was inactive, while F211W/N327D produced only trace amounts of sesquiterpenes. It is notable, however, that other than the uncyclized farnesol, Gd4ol was the most abundant product in this variant, albeit at <1.0 mg/L_org_ (Figure 3A).

Instead, we attempted to engineer the Gd4ol synthase using a different approach. F104 is on the opposite side of the active site to N327, with its phenyl ring well placed to stabilize C7 of cation C by cation−π interactions (Figure 2D). According to our proposed scheme (Figure 1), a positive charge accumulates on C7 in cation C immediately after the intramolecular cyclization of cation B. If the conversion of cation A to B to C is a key step to progressing beyond the germacrane scaffold, then destabilizing cation C could have a similar effect on product profile as did stabilizing cations A and B in our N327D variant.

F104A produced trace amounts of cyclic sesquiterpenes, not including 10-*epi*-cubebol, consistent with F104 shaping the product-like active site contour. F104L produced very small amounts of 10-*epi*-cubebol (0.38 mg/L_org_). Intriguingly, this variant also produced 1.23 mg/L_org_ of Gd4ol, suggesting that disturbing the interaction between ScCubS and cation C pushes the distribution back in favor of cation A, as predicted. F104Y was the most successful variant, producing 10-*epi-*cubebol at 1.23 mg/L_org_, while making Gd4ol at 7.59 mg/L_org_, representing ∼80% of all sesquiterpenes detected, and effectively making F104Y a germacradien-4-ol synthase (Figure 3A). Restoration of the aromatic side chain in F104Y provides a better active site contour, but the weak plasticity between phenylalanine and tyrosine impairs ScCubS’s ability to stabilize C7, driving the equilibrium in favor of cations A and B. The disrupted active site is perhaps due to changes in the local hydrogen-bonding network caused by the additional hydroxyl group on tyrosine, as proposed elsewhere for similar results.^30^ This leads us to postulate that in ScCubS, the substrate undergoes ambient hydroxylation, perhaps by a water molecule hydrogen-bonded to N327 and/or N248 and that the final quenching product depends on the lifetime of the various cations sampled throughout the reaction mechanism, which is controlled by residues such as N327 and F104.

### F211 Unlocks the Third and Final Cyclization

Once cation C has been achieved by the second cyclization, a similar situation arises. ScCubS must once again prevent quenching, allowing the conversion of cation C first to cation D and ultimately to cation E, the precursor to 10-*epi*-cubebol.

For F211A the cubebane, 10-*epi*-cubebol was replaced as the major product by the cadalane 1,10-di-*epi*-cubenol, derived from cation D (Figure 1). In the WT enzyme, cation D undergoes intramolecular cyclization to give cation E, leaving a positive charge at C3, which is quenched by water to give 10-*epi*-cubebol. 1,10-di-*epi*-cubenol, however, is delivered by hydroxylation of cation D at C6, suggesting a role for F211 in promoting the conversion of cation D to cation E, and subsequently to 10-*epi*-cubebol. F211 is positioned “above” the substrate according to the scheme shown in Figure 1, and C7 is sandwiched between F211 and F104 (Figure 2D). Hydroxylation to give 1,10-di-*epi*-cubenol also occurs from above, whereas the intramolecular ring closure which delivers cation E occurs from below. We also detected relatively high quantities of two other cadalanes in F211A, γ-cadinene, and τ-cadinol (Figures S2 and S33), which result from the deprotonation and hydroxylation of cation C, respectively. All of this strongly suggests that F211 helps to block hydroxylation (or deprotonation) of cations C and D that would quench the reaction and permits the formation of cation E which continues the cascade. It also very likely plays a supplementary role in stabilizing the positive charge on C6 and/or C7 in cations C and D, as shown by the improved product profiles in our aromatic F211 variants (Table S2).

F211L restored 10-*epi*-cubebol as the major product, but at only 2.66 mg/L_org_. Interestingly, 1,10-di-*epi*-cubenol was not detected in this variant. Although unable to offer cation−π stabilization, the greater size of leucine versus alanine is seemingly enough to block the premature hydroxylation or deprotonation described above, further supporting F211’s role in this part of the reaction.

Restoring aromaticity in the F211Y and F211W variants delivered 10-*epi*-cubebol as the main product at yields of 4.77 and 11.59 mg/L, respectively. Both of these mutants can presumably stabilize C6/C7 and block quenching, albeit less well than phenylalanine. This again points toward the choice of phenylalanine at position F211 being highly specific, which is supported by its high conservation in certain TSs even versus other residues in the aromatic family.^31^ Preventing the quenching of cations C and D, and stabilizing C6 and C7 of these same cations, is key to unlocking the third cyclization step needed to produce 10-*epi*-cubebol.

### Serine as the Effector Residue

The “PPi sensor” R202 is linked to S206 via T205, and the carbonyl of S206 is well placed to interact with C3 of FPP. This is consistent with a previously established induced-fit mechanism for FPP ionization and isomerization,^25^ and the now-established role of carbonyls in stabilizing carbocations in TS,^29^ which was recently quantified for a diterpene synthase benefitting from a highly relevant crystal structure.^8^ Upon sensing the PPi of FPP, R202 brings the “effector” residue S206 close to C3 of FPP via the “linker” residue T205, whereupon a lone pair from the main-chain S206 carbonyl donates electron density into the π* molecular orbital of the C2,3 double bond, helping to trigger ionization. After ionization, the S206 carbonyl presumably helps stabilize the positive charge at the allylic C2,3 position. In bacterial TSs, the most common linker is threonine, as observed here. The effector residue is overwhelmingly glycine, while alanine, valine, and serine are the other, much less common, choices^25^ (Figures S5 and S6). To understand the role of S206 in ScCubS, we created S206 variants that contained the more common effectors. We also created a bulky variant, S206F, and two variants chemically similar to the WT—S206C and S206T.

All S206 variants showed reduced 10-*epi*-cubebol titers versus the WT, suggesting that the choice of serine is important. Introducing significant steric bulk in the S206F variant produced an inactive enzyme. S206 and its neighbor T205 occupy the so-called “kink region” of the G-helix of TSs,^25,32^ a short, flexible region that moves toward the substrate in response to PPi being sensed by R202 (Figure 2C). The absence of any products in S206F (including those from the shorter chain GPP) is consistent with the presumed role of S206 in stabilizing the initial isomerization of the farnesyl cation to the nerolidyl cation, and perhaps the ability of ScCubS to sequester its substrate from bulk solution. S206T produced trace amounts of 10-*epi*-cubebol, meaning a single additional methyl group on the side chain was poorly tolerated. S206C, which represents a change of a single atom (oxygen to sulfur), produced 10-*epi*-cubebol at 1.45 mg/L_org_. The chemical similarity of sulfur to oxygen, and the dramatic difference in product profile observed in these S206 variants, led us to believe that the side-chain hydroxyl group in S206 was important for 10-*epi*-cubebol synthesis.

**Figure 4:**
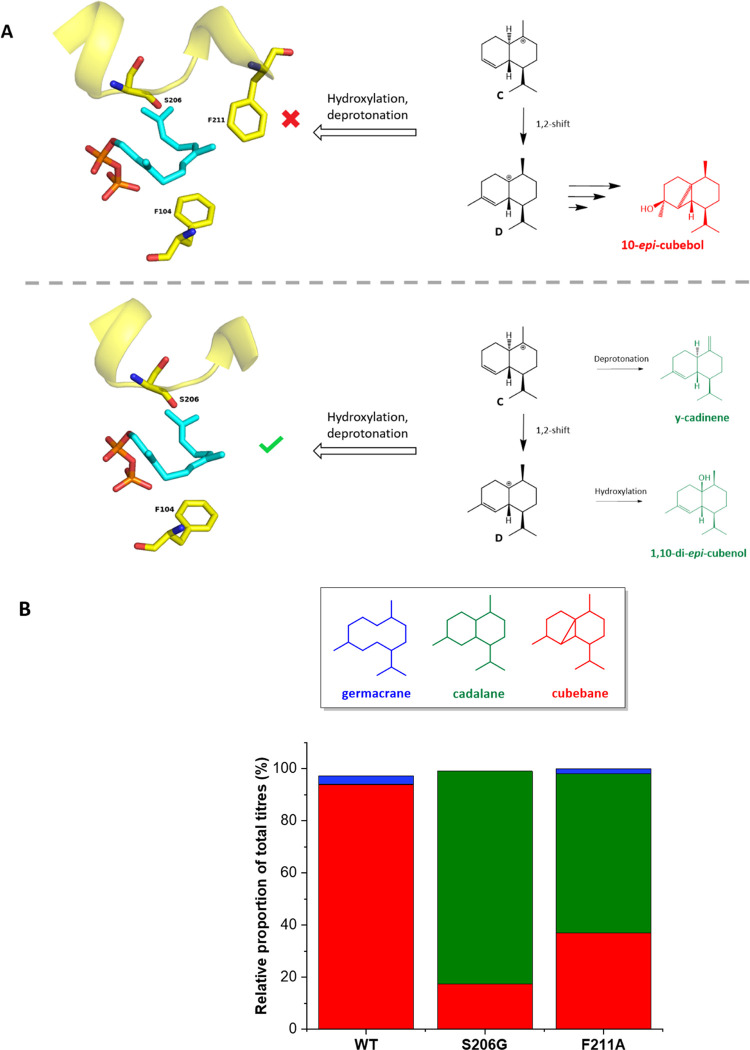
F211 blocks premature quenching. (A) In the wild-type F211 blocks the quenching of cations C and D. When F211 cannot be properly enlisted (S206G) or is mutated to a smaller residue (F211A), premature quenching occurs and multiple cadalane compounds are produced, including some not observed in the wild type (Figure S2). (B) Relative proportions of germacrane, cadalane, and cubebane compounds in the WT, S206G, and F211A variants. The WT makes very few cadalanes, while S206G, and F211A make mostly cadalanes. The different hydrocarbon scaffolds are shown in the top box.

Similar results were observed for the more common effector residues. S206V was inactive, while S206A produced 10-*epi-*cubebol at 5.37 mg/L_org_, representing an almost 10-fold decrease vs the WT enzyme. Most strikingly, replacing S206 with the most common effector, glycine, resulted in a promiscuous enzyme that produced mostly cadalanes, including several not detected with the WT enzyme. Moreover, most of these were more abundant than 10-*epi*-cubebol, effectively making S206G a cadalane-type synthase (Figures 4B and S2).

It is striking that the mechanistic implications of mutating S206 should be the same as mutating F211. The small, polar side chain of S206 points away from the active site; the bulky, nonpolar side chain of F211 points directly at the substrate. And yet, the results obtained with the S206 variants show that S206, like F211, plays a fundamental role in achieving the final cubebane hydrocarbon scaffold.

The crystal structure for ScCubS-Mg_3_^2+^-PPi and our docking models reveal that the hydroxyl side chain of S206 is perfectly positioned to form a hydrogen bond (3.1 Å between heteroatoms) to the amine of the nearby M208 (Figure 2C) which is, in turn, hydrogen-bonded to F211. This places S206 at the center of hydrogen-bonding network on the flexible and catalytically important G-helix. We conclude that this hydrogen-bonding network helps S206 enlist F211 as an extension of the G-helix response to sensing FPP, sandwiching C7 between F211 and F104, blocking quenching, and providing the stabilization needed to drive the reaction to completion. Disrupting this hydrogen-bonding network, either with changes in electronegativity or steric orientation, diminishes this concerted action, affecting the active site contour and preventing the third and final cyclization step required to produce 10-*epi*-cubebol. Although much less common, it appears that in ScCubS the presence of serine as the effector residue is critical, and could represent yet another important evolutionary expansion in the capabilities of TSs.

## Conclusions

We determined the crystal structure of ScCubS in complex with a trinuclear magnesium cluster and pyrophosphate, and used a combined computational and experimental approach to understand the mechanism of this enzyme. At each stage of 10-*epi*-cubebol synthesis, multiple competing pathways must be blocked, and the main branch stabilized to ensure delivery of the final terpenoid product.

The conversion of cation B to C and subsequently D to E, the second and third intramolecular cyclization steps, are key to producing cubebanes instead of germacranes or cadalanes. In ScCubS, the residues described in this work act in concert to ensure the reaction cascade continues through to 10-*epi*-cubebol without being prematurely quenched. This is consistent with altered product profiles in other TSs, which are in most cases the result of premature quenching of the reaction due to a relaxed control over the carbocation intermediates.^14,33,34^

Cations A and B are the parent cations of the germacranes produced by ScCubS, while cations C and D result in cadalanes. N327 and F104 drive the substrate beyond the germacrane scaffold and open up the cadalanes by unlocking the second intramolecular cyclization. F104Y illustrated this dramatically by acting as a Gd4ol synthase. S206 and F211 then unlock the third cyclization which achieves cubebanes, including 10-*epi*-cubebol. Both F211A and S206G acted as cadalane-type synthases.

Each of these cyclization-enabling motifs potentially represents an important evolutionary expansion in the chemical space accessible by TSs. The rational design of enzymes depends on identifying and understanding these fundamentally important events. By tackling this complex, promiscuous enzyme, we have provided important new insights that will assist in the future engineering of other terpene synthases.

## Supporting Information Available

Experimental section; product profiles; crystallography data; structure and sequence alignments; gas chromatography–mass spectrometry (GC–MS) analysis, and density functional theory (DFT) calculations (PDF)
