# Supplementary material for: How a 10-epi-Cubebol Synthase Avoids Premature Reaction Quenching to Form a Tricyclic Product at High Purity
Source: ACS Catal. 2022 Sep 21;12(19):12123–31. doi: 10.1021/acscatal.2c03155 (PMC9552170; doi:10.1021/acscatal.2c03155)
Supplement: Supplementary file 1 — cs2c03155_si_001.pdf [file cs2c03155_si_001.pdf]

Supporting information

How a 10-*epi*-cubebol Synthase Avoids Premature Reaction Quenching to Form a Tricyclic Product at High Purity

Joshua N. Whitehead[1$], Nicole G. H. Leferink[2$], Gajendar Komati Reddy[1$], Colin W. Levy[1], Sam Hay[1], Eriko Takano[1,2,*] and Nigel S. Scrutton[1,2,*]

[1]Manchester Institute of Biotechnology, Department of Chemistry, the University of Manchester, Manchester, M1 7DN, United Kingdom.

[2]Future Biomanufacturing Research Hub (FBRH), Manchester Institute of Biotechnology, Department of Chemistry, The University of Manchester, Manchester, M1 7DN, United Kingdom.

* Corresponding authors: nigel.scrutton@manchester.ac.uk, eriko.takano@manchester.ac.uk

Table of Contents

Experimental section ... 3

Expression and purification of 10-*epi*-cubebol synthase and variants ... 3

Site-directed mutagenesis ... 3

Plasmids used in this study ... 6

Crystallisation and structure determination of 10-*epi*-cubebol synthase ... 7

Structure solution ... 7

Molecular Docking ... 9

DFT calculations ... 9

GC-MS Analysis ... 9

Product profiles for all ScCubS variants ... 10

Crystal structure analysis ... 13

Mechanism of 10-*epi*-cubebol formation ... 15

Choosing the conformation of FPP ... 17

DFT calculations and docking of intermediates ... 17

Sequence alignment and the common effector residues in bacterial terpene synthases ... 19

GC and GCMS analysis ... 20

Chemicals and standards ... 20

Mass spectra for identified sesquiterpenes ... 22

GCMS chromatograms for variants ... 31

References ... 39

Experimental section

Expression and purification of 10-*epi*-cubebol synthase and variants

The full-length gene encoding for 10-*epi*-cubebol synthase (ScCubS; sce6369, CAN96536.1, A9GK58)[1,2] from *Sorangium cellulosum* So ce 56 was optimized for codon usage, synthesized and subcloned into a pETM11 vector digested with NcoI and XhoI by GeneArt (Life Technologies). The final construct codes for 10-*epi*-cubebol synthase with a His_6 tag followed by a TEV protease cleavage site at the N-terminus. For expression and purification of protein the plasmid was transformed into *E. coli* BL21 (DE3) cells (NEB), and a single colony was inoculated into 50 mL of 2XYT media containing 50 µg/mL of kanamycin and grown overnight at 37°C. The culture was diluted into 3 L of fresh 2X-YT media containing 50 µg/mL of kanamycin and allowed to grow at 37°C until the OD_{600nm} reached 0.6−0.8. After inducing with 0.05 mM Isopropyl β-D-1-thiogalactopyranoside (IPTG) the temperature was reduced to 16°C and the incubation was continued for 14−18 h. The cells were centrifuged at 6000g for 10 min, and the pellet was resuspended in buffer A (50 mM Tris-HCl pH 7.5, 500 mM NaCl, 1 mM Tris (2-chloroethyl) phosphate (TCEP) and 5 mM $MgCl_2$). The cells were sonicated on ice using a 50 % duty cycle at 50 % power for 5 mins, and the debris was removed by centrifugation at 40,000 g for 30 min. The supernatant was filtered through a 0.2 µm filter and loaded onto a 5 mL HisTrap column (GE Healthcare) pre-equilibrated with buffer A. The column was washed with buffer A containing 10 mM imidazole (pH 8.0) and increased to 40 mM imidazole by step gradients with 5 column volume for each concentration. Increasing the concentration of imidazole to 100-250 mM eluted the protein. The purified protein was desalted using a Centripure P100 column (emp Biotech GmbH) equilibrated with buffer B containing 50 mM Tris-HCl, 150 mM NaCl, 5 mM $MgCl_2$ and 1 mM TCEP. His-tagged protein was concentrated and loaded onto a Hiload Superdex (26/60) S75 column (GE Healthcare) pre-equilibrated with buffer B and pure fractions corresponding to the molecular weight of ≈80 kDa collected and 10 mg/mL protein used for crystal setup.

Site-directed mutagenesis

A total of 17 new ScCubS single-point mutants and two double-point mutants were introduced via the polymerase chain reaction (PCR). Forward and reverse primers (Table S1)

were used to introduce mutations into the wild-type ScCubS plasmid. Two different methods were used for different variants, as shown in Table S1. For method 1: deionized H_2O (31.5 µL), 5× Q5 reaction buffer (10 µL), 10 mM dNTPs (1 µL), 10 µM forward/reverse primer mix (1.5 µL), 50-150 ng/µL wild-type ScCubS template plasmid (5 µL), and Q5 Hot Start DNA Polymerase (1 µL) were combined to give a total reaction volume of 50 µL. For PCR amplification the following cycle was used: initial denaturation at 98°C (2 min), 18x cycles of: denaturation at 98°C (30 s), annealing at 55-68 °C (60 s), and extension at 72°C for 30 s/kb, then after 18x cycles a final extension at 72°C (10 min). The PCR mixture was incubated at 37 °C for a minimum of 3 h with FastDigest DpnI (1 µL). The PCR product (2.5 - 4.0 µL) was transformed into 25 µL DH5a cells. Plasmid DNA was extracted from single colonies with a QIAprep Spin Miniprep Kit and point mutation was confirmed by standard Sanger sequencing.

For method 2: deionized H_2O (35.5 µL), 5× Phusion HF buffer (10 µL), 10 mM dNTPs (1 µL), 0.5 µM forward primer (1 µL), 0.5 µM reverse primer (1 µL), 50 ng/µL wild-type ScCubS template plasmid (1 µL), and 0.02 U/µL Phusion Hot Start DNA Polymerase (0.5 µL) were combined to give a total reaction volume of 50 µL. For PCR amplification the following cycle was used: initial denaturation at 98°C (30 s), 18x cycles of: denaturation at 98°C (10 s), annealing at 60-65 °C (30 s), and extension at 72°C (30 s/kb), then after 18x cycles a final extension at 72°C (10 min). The PCR mixture was incubated at 37 °C for 2 h with FastDigest DpnI (1 µL). PCR product (20 ng) was circularised using T4DNA ligase for 5 minutes as manufacturer advised and transformed 2 uL into DH5a cells. Plasmid DNA was extracted from single colonies with a QIAprep Spin Miniprep Kit and point mutation was confirmed by standard Sanger sequencing.

Table S1: List of oligonucleotides used for mutagenesis in this study. Altered base pairs are in lower case.

**Primer name**	**Sequence (5'-3')**	**Method**
F104A_fwd	GATTATTACAGCTGGCTGgccTTCTTCGATGATGTTTGTG	1
F104A_rev	CACAAACATCATCGAAGAAggcCAGCCAGCTGTAATAATC	1
F104L_fwd	GATTATTACAGCTGGCTGcTgTTCTTCGATGATGTTTGTG	1
F104L_rev	CACAAACATCATCGAAGAAcAgCAGCCAGCTGTAATAATC	1

F104Y_fwd	GATTATTACAGCTGGCTGTaCTTCTTCGATGATGTTTGTG	1
F104Y_rev	CACAAACATCATCGAAGAAGtACAGCCAGCTGTAATAATC	1
S206A_fwd	GGTATGCGCATGCATACCgccACCATGTATGAATTTTGGG	2
S206A_rev	CCCAAAATTCATACATGGTggcGGTATGCATGCGCATACC	2
S206C_fwd	GGTATGCGCATGCATACCtgcACCATGTATGAATTTTGGG	2
S206C_rev	CCCAAAATTCATACATGGTgcaGGTATGCATGCGCATACC	2
S206G_fwd	GGTATGCGCATGCATACCgGCACCATGTATGAATTTTGGG	1
S206G_rev	CCCAAAATTCATACATGGTGCcGGTATGCATGCGCATACC	1
S206V_fwd	GGTATGCGCATGCATACCgtCACCATGTATGAATTTTGGG	1
S206V_rev	CCCAAAATTCATACATGGTGacGGTATGCATGCGCATACC	1
S206F_fwd	CATACATGGTaaaGGTATGCATGCGCATACCTTC	2
S206T_fwd	ATACATGGTggtGGTATGCATGCGCATACCTTC	2
S206_rev	GAATTTTGGGATTTTATCGAATATGCC	2
F211A_Fwd	ATAAAATCCCAcgcTTCATACATGGTGCTGGTATG	2
F211L_fwd	ATAAAATCCCAcagTTCATACATGGTGCTGGTATG	2
F211W_fwd	ATAAAATCCCAccaTTCATACATGGTGCTGGTATG	2
F211_rev	CGAATATGCCGGTGACCTGTTTC	2
F211Y_rev2	TTCGATAAAATCCCAataTTCATACATGGTGCTGG	2
F211Y_fwd2	TATGCCGGTGACCTGTTTCTGCC	2
A244F_fwd	CGTCGTGCAGGTAATGCAATTttCAGCTTTGCAAATGATATC	1
A244F_rev	GATATCATTTGCAAAGCTGaaAATTGCATTACCTGCACGACG	1
A244G_fwd	CGTCGTGCAGGTAATGCAATTGgCAGCTTTGCAAATGATATC	1
A244G_rev	GATATCATTTGCAAAGCTGcCAATTGCATTACCTGCACGACG	1
N327A_fwd	GTATTCGTATTTGGATTCGTGCAgcgCATGATTGGAGCATTGTTAC	1
N327A_rev	GTAACAATGCTCCAATCATGcgcTGCACGAATCCAAATACGAATAC	1
N327D_fwd	GTATTCGTATTTGGATTCGTGCAgACCATGATTGGAGCATTGTTAC	1
N327D_rev	GTAACAATGCTCCAATCATGGTcTGCACGAATCCAAATACGAATAC	1
N327D/F211L	n/a	1
N327D/F211W	n/a	1

Plasmids used in this study

Table S2: List of plasmids used in this study

Plasmid reference	Plasmid name	Description	Source
pMVA	pBbA5a-MTSAe-T1f-MBI(f)- T1002i	p15A, Kanr, PlacUV5, MTSA, T1, MBI-f, T1002	[3]
pBbB2a-GPPS-CAN96536	pBbB2a-trAgGPPS(co)-ScCubS	pBBR, Ampr, Ptet, trAgGPPS(co)- ScCubS	[2]
pBb-GPPS-ScCubS-F104A	pBbB2a-trAgGPPS(co)-ScCubS-F104A	pBBR, Ampr, Ptet, trAgGPPS(co)-ScCubS-F104A	This study
pBb-GPPS-ScCubS-F104L	pBbB2a-trAgGPPS(co)-ScCubS-F104L	pBBR, Ampr, Ptet, trAgGPPS(co)-ScCubS-F104L	This study
pBb-GPPS-ScCubS-F104Y	pBbB2a-trAgGPPS(co)-ScCubS-F104Y	pBBR, Ampr, Ptet, trAgGPPS(co)-ScCubS-F104Y	This study
pBb-GPPS-ScCubS-S206A	pBbB2a-trAgGPPS(co)-ScCubS-S206A	pBBR, Ampr, Ptet, trAgGPPS(co)-ScCubS-S206A	This study
pBb-GPPS-ScCubS-S206C	pBbB2a-trAgGPPS(co)-ScCubS-S206C	pBBR, Ampr, Ptet, trAgGPPS(co)-ScCubS-S206C	This study
pBb-GPPS-ScCubS-S206F	pBbB2a-trAgGPPS(co)-ScCubS-S206F	pBBR, Ampr, Ptet, trAgGPPS(co)-ScCubS-S206F	This study
pBb-GPPS-ScCubS-S206G	pBbB2a-trAgGPPS(co)-ScCubS-S206G	pBBR, Ampr, Ptet, trAgGPPS(co)-ScCubS-S206G	This study
pBb-GPPS-ScCubS-S206T	pBbB2a-trAgGPPS(co)-ScCubS-S206T	pBBR, Ampr, Ptet, trAgGPPS(co)-ScCubS-S206T	This study
pBb-GPPS-ScCubS-S206V	pBbB2a-trAgGPPS(co)-ScCubS-S206V	pBBR, Ampr, Ptet, trAgGPPS(co)-ScCubS-S206V	This study
pBb-GPPS-ScCubS-F211A	pBbB2a-trAgGPPS(co)-ScCubS-F211A	pBBR, Ampr, Ptet, trAgGPPS(co)-ScCubS-F211A	This study
pBb-GPPS-ScCubS-F211L	pBbB2a-trAgGPPS(co)-ScCubS-F211L	pBBR, Ampr, Ptet, trAgGPPS(co)-ScCubS-F211L	This study
pBb-GPPS-ScCubS-F211W	pBbB2a-trAgGPPS(co)-ScCubS-F211W	pBBR, Ampr, Ptet, trAgGPPS(co)-ScCubS-F211W	This study
pBb-GPPS-ScCubS-F211Y	pBbB2a-trAgGPPS(co)-ScCubS-F211Y	pBBR, Ampr, Ptet, trAgGPPS(co)-ScCubS-F211Y	This study
pBb-GPPS-ScCubS-A244F	pBbB2a-trAgGPPS(co)-ScCubS-A244F	pBBR, Ampr, Ptet, trAgGPPS(co)-ScCubS-A244F	This study
pBb-GPPS-ScCubS-A244G	pBbB2a-trAgGPPS(co)-ScCubS-A244G	pBBR, Ampr, Ptet, trAgGPPS(co)-ScCubS-A244G	This study
pBb-GPPS-ScCubS-N327A	pBbB2a-trAgGPPS(co)-ScCubS-N327A	pBBR, Ampr, Ptet, trAgGPPS(co)-ScCubS-N327A	This study
pBb-GPPS-ScCubS-N327D	pBbB2a-trAgGPPS(co)-ScCubS-N327D	pBBR, Ampr, Ptet, trAgGPPS(co)-ScCubS-N327D	This study
pBb-GPPS-ScCubS-N327D/F211L	pBbB2a-trAgGPPS(co)-ScCubS-N327D/F211L	pBBR, Ampr, Ptet, trAgGPPS(co)-ScCubS-N327D/F211L	This study
pBb-GPPS-ScCubS-N327D/F211W	pBbB2a-trAgGPPS(co)-ScCubS-N327D/F211W	pBBR, Ampr, Ptet, trAgGPPS(co)-ScCubS-N327D/F211W	This study

Crystallisation and structure determination of 10-*epi*-cubebol synthase

ScCubS was crystallised by sitting drop vapour diffusion using drops comprised of 200nl of 10mg/ml 10-*epi*-cubebol synthase mixed with an equal volume of reservoir solution. Plates were incubated at 4°C for a period of 6 days. Crystals of ScCubS grew in the presence of 0.1 M Imidazole; MES monohydrate (acid) pH – 6.5, 0.03 M of each divalent cation, and 30% precipitant mix 2 (40% v/v Ethylene glycol; 20% w/v PEG 8000) [Morpheus A2 Molecular Dimensions]. The ScCubS crystals were soaked with 20 mM trisammonium (2Z,6E)-2-fluorofarnesylpyrophosphate for 3-4 hours prior to crycooling. Trisammonium (2Z,6E)-2-fluorofarnesylpyrophosphate was synthesised and characterised by the previously reported procedure[18], as shown in Scheme S1.

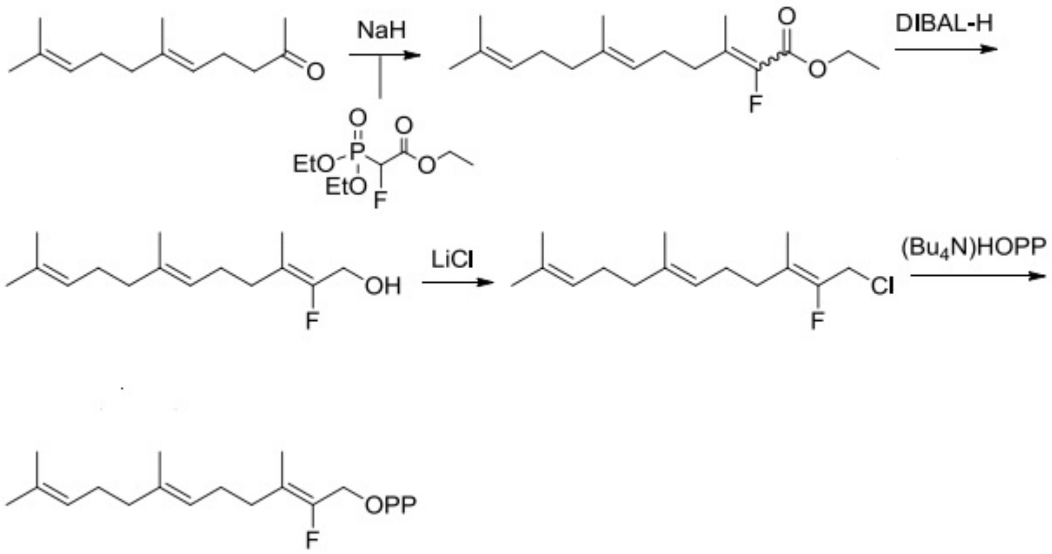

Scheme S1: Synthesis of trisammonium (2Z,6E)-2-fluorofarnesylpyrophosphate.

Structure solution

Single crystal data were collected at Diamond Light Source for a (2Z,6E)-2-fluorofarnesylpyrophosphate (FPP) complex of ScCubS, subsequent structure determination of this data resulted in a PPi-complexed ScCubS. Data reduction was performed in Dials and subsequent structure determination achieved through molecular replacement in Phaser with a search model derived from Streptomyces exfoliates pentalenene synthase (1PS1). Iterative

cycles of model building and refinement were performed in COOT and Phenix.refine respectively. Validation with MOLPROBITY and PDBREDO were integrated into the iterative rebuild and refinement process. Data collection and refinement statistics are shown in Table S3. The atomic coordinates and structure factors have been deposited in the Protein Data Bank with accession code 7ZRN.

Table S3. Data collection and refinement statistics for ScCubS-PPi complex

**Wavelength**	0.9159
**Resolution range**	66.92 - 1.82 (1.885 - 1.82)
**Space group**	P 21 21 21
**Unit cell**	74.315 89.644 100.563 90 90 90
**Total reflections**	376858 (31120)
**Unique reflections**	60876 (5964)
**Multiplicity**	6.2 (5.2)
**Completeness (%)**	99.92 (99.72)
**Mean I/sigma(I)**	9.36 (1.08)
**Wilson B-factor (Å^2)**	25.10
**R-merge**	0.1151 (1.384)
**R-meas**	0.1257 (1.54)
**R-pim**	0.04997 (0.6683)
**CC1/2**	0.998 (0.506)
**CC***	1 (0.82)
**Reflections used in refinement**	60839 (5962)
**Reflections used for R-free**	2000 (196)
**R-work**	0.1787 (0.2878)
**R-free**	0.2060 (0.3272)
**CC(work)**	0.946 (0.684)
**CC(free)**	0.935 (0.639)
**Number of non-hydrogen atoms**	5384
**macromolecules**	4951
**ligands**	24
**solvent**	409
**Protein residues**	601
**RMS(bonds, Å)**	0.009
**RMS(angles, °)**	1.09
**Ramachandran favoured (%)**	99.32
**Ramachandran allowed (%)**	0.51
**Ramachandran outliers (%)**	0.17
**Rotamer outliers (%)**	0.00
**Clash score**	5.76
**Average B-factor (Å^2)**	29.54
**macromolecules**	28.93
**ligands**	28.38
**solvent**	26.82
**Number of TLS groups**	1

Statistics for the highest-resolution shell are shown in parentheses.

Molecular Docking

In the absence of an ScCubS-FPP complex, molecular docking of FPP was performed in ICM Pro (Molsoft). The ScCubS-PPi model was used as the starting point of the docking with additional positional restraints to the PPi position imposed during the simulation. The top docking result is presented in Figure S3.

Molecular docking of geometry optimised intermediates was performed using AutoDock Vina. The ScCubS-PPi model acted as the receptor, and each intermediate was docked with conformational freedom according to its valency. A search space of 12 x 12 x 14 Å was manually defined to include the active site, and the top 8 conformers were ordered according to AutoDock's binding score. In all three cases the docking conformer shown in Figure S4 was one of the top 8 results.

DFT calculations

Intermediates B, C and the germacradiene-4-ol were modeled from the docked FPP structure using DFT at the B3LYP/6-31+G(d,p) level of theory with implicit water solvation using the PCM model implemented in Gaussian 16 rev C.01. These were aligned with the docked FPP structure using Pymol's align command on selected atoms.

Analysis of product diversity generated by 10-*epi*-cubebol synthase

For terpenoid production, the pBbB2a-GPPS-ScCubS and ScCubS mutant plasmids were co-transformed with pMVA into *E. coli* DH5α and grown as described before.[2] Expression strains were inoculated in phosphate buffered Terrific Broth (TB) supplemented with 0.4% glucose in glass screw capped vials, and induced for 48 h at 30 °C, 200 rpm with 50 µM IPTG and 25 nM anhydro-tetracycline (aTc). A 20 % (v/v) n-nonane layer was added to capture the volatile terpenoid products. After induction, the nonane overlay was collected, dried over anhydrous $MgSO_4$, and spiked with 0.01% (v/v) sec-butyl benzene as internal standard.

GC-MS Analysis

Samples were injected onto an Agilent Technologies 7890B GC equipped with an Agilent Technologies 5977A MSD. The products were separated on a DB-WAX column (30 m × 0.32 mm i.d., 0.25 µm film thickness, Agilent Technologies). The injector temperature was set at 240 °C with a split ratio of 20:1 (1 µL injection). The carrier gas was helium with a flow rate of

1 mL/min and a pressure of 5.1 psi. The following oven program was used: 50 °C (1 min hold), ramp to 68 °C at 5 °C/min (2 min hold), and ramp to 230 °C at 25 °C/ min (2 min hold). The ion source temperature of the mass spectrometer (MS) was set to 230 °C, and spectra were recorded from m/z 50 to m/z 250. Compound identification was carried out using authentic standards and/or by comparison to reference spectra in the NIST library of MS spectra and fragmentation patterns as described previously. The retention times and order of elution were compared to the previously characterised WT product profile of ScCubS.[1] Terpenoids were quantified using authentic standards wherever possible, using experimentally determined relative response factors in relation to the internal standard. Where standards were not available, titres for these compounds were estimated by comparing the relevant ion count against the average of the response for farnesol and nerolidol.

Product profiles for all ScCubS variants

Table S4: Titres for detected sesquiterpenoid products in all ScCubS variants. Average titres are calculated from a minimum of 2-6 replicates, and are given in mg/L of organic overlay.

**Variant**	**Product**	**Average titre (mg/L_{org})**	**± SD**
**WT**	10-*epi*-cubebol	43.40	14.33
	(*E*)-β-farnesene	0.61	0.16
	ST1	0.33	0.15
	Germacrene D	0.71	0.20
	ST2	0.85	0.08
	γ-cadinene	0.84	0.63
	ST4	0.52	0.19
	Cubebol	2.03	0.45
	ST5	0.09	0.13
	Germacradien-4-ol	1.03	0.31
	Cis-muurola-3,5-diene	0.14	0.10
	ST6	3.52	4.97
**S206A**	10-*epi*-cubebol	5.37	3.24
	Germacrene D	0.05	0.06
	Germacradien-4-ol	0.12	0.17
**S206C**	10-*epi*-cubebol	1.45	0.93
	Cubebol	0.15	0.21
**S206G**	α-cubebene	14.77	11.74
	Cis-muurola-3,5-diene	15.23	12.31
	Cadina-3,5-diene	10.29	8.71
	(*E*)-β-farnesene	1.05	1.49

	Cis-muurola-4,5-diene	10.78	9.21
	Epizonarene	3.74	2.51
	Cadina-1,4-diene	21.76	12.08
	γ-elemene	0.64	0.91
	Cadina-3,9-diene	22.39	18.63
	γ-cadinene	2.39	3.37
	α-cadinene	1.05	1.48
	10-*epi*-cubebol	4.16	5.89
	1,10-di-*epi*-cubenol	1.00	1.41
**S206F**	Inactive		
**S206T**	10-*epi*-cubebol	0.06	0.08
**S206V**	Inactive		
**F211A**	γ-cadinene	0.67	0.07
	10-*epi*-cubebol	0.96	0.05
	Cubebol	0.48	0.02
	1,10-di-*epi*-cubenol	1.28	0.05
	τ-cadinol	0.43	0.07
	Germacradien-4-ol	0.08	0.11
**F211L**	(*E*)-β-farnesene	0.14	0.10
	10-*epi*-cubebol	2.66	0.45
	Germacradien-4-ol	0.66	0.11
	α-cubebene	0.05	0.07
	Nerolidol	0.06	0.08
**F211Y**	10-epi-cubebol	4.77	1.11
	Cubebol	0.18	0.04
	Germacradien-4-ol	0.14	0.10
**F211W**	(*E*)-β-farnesene	0.25	0.04
	Germacrene D	0.37	0.11
	10-*epi*-cubebol	11.59	2.38
	Cubebol	0.26	0.07
	Nerolidol	0.18	0.06
	Germacradien-4-ol	0.96	0.44
	α-cubebene	0.04	0.06
	ST2	0.12	0.09
	Cis-muurola-4,5-diene	0.04	0.06
**A244F**	Inactive		
**A244G**	(*E*)-β-farnesene	0.26	0.04
	ST2	0.22	0.03
	Cis-muurola-4,5-diene	0.18	0.02
	ST1	0.16	0.02
	Germacrene D	0.41	0.04
	ST4	0.16	0.01
	10-*epi*-cubebol	17.15	0.93

	Cubebol	0.74	0.05
	Germacradien-4-ol	0.70	0.07
	1,10-di-*epi*-cubenol	0.28	0.01
	ST3	0.12	0.09
	α-cubebene	0.10	0.07
	ST5	0.17	0.13
**N327A**	10-*epi*-cubebol	0.20	0.02
	Germacradien-4-ol	0.15	0.11
**N327D**	ST4	0.19	0.03
	10-*epi*-cubebol	5.47	1.28
	Cubebol	0.61	0.60
	Germacradien-4-ol	2.95	0.96
	Germacrene D	0.13	0.09
	ST2	0.25	0.18
	(*E*)-β-farnesene	0.06	0.08
	Cis-muurola-4,5-diene	0.05	0.07
**F211L/N327D**	Inactive		
**F211W/N327D**	10-*epi*-cubebol	0.05	0.07
	Germacradien-4-ol	0.19	0.16
	Farnesol	1.55	1.33
**F104A**	Germacrene D	0.27	0.40
	γ-cadinene	0.12	0.18
	Nerolidol	1.23	1.55
	Farnesol	2.16	3.24
**F104L**	10-*epi*-cubebol	0.38	0.39
	ST4	0.33	0.46
	Germacradien-4-ol	1.23	1.05
	Farnesol	2.05	1.50
**F104Y**	10-*epi*-cubebol	1.23	0.44
	γ-cadinene	1.12	0.39
	Cubebol	0.36	0.15
	Germacradien-4-ol	7.59	2.06

Crystal structure analysis

The structure of the ScCubs-Mg^{2+}_3-PPi complex was determined at 1.80 Å resolution, and reveals that ScCubS crystallises as a homodimer. ScCubS exhibits the classical class I TS structural features, comprising a bundle of 17 α-helices and a central hydrophobic cavity. ScCubS is structurally similar to other solved bacterial sesquiterpene synthases (Figure S2), including epi-isozizaene synthase (EIZS), which crystallises as a monomer.[10] The electron density map of ScCubs shows the PPi bound in the active site, coordinated to the trinuclear Mg^{2+} custer, similar to other known TS structures.[10-12] The side chain of D107 in the aspartate-rich $D^{107}DVCE^{111}$ motif coordinates to Mg^{2+}_A and Mg^{2+}_C with syn, syn-bidentate geometry, while Mg^{2+}_B is chelated by the $\mathbf{N}^{248}\mathbf{D}^{249}IY\mathbf{S}^{252}LRK\mathbf{E}^{256}$ motif (bold indicates Mg^{2+} ligands), similar to EIZS. Each Mg^{2+} ion is coordinated with octahedral geometry, with non-protein coordination sites occupied by the oxygen atoms of PPi and by water molecules.

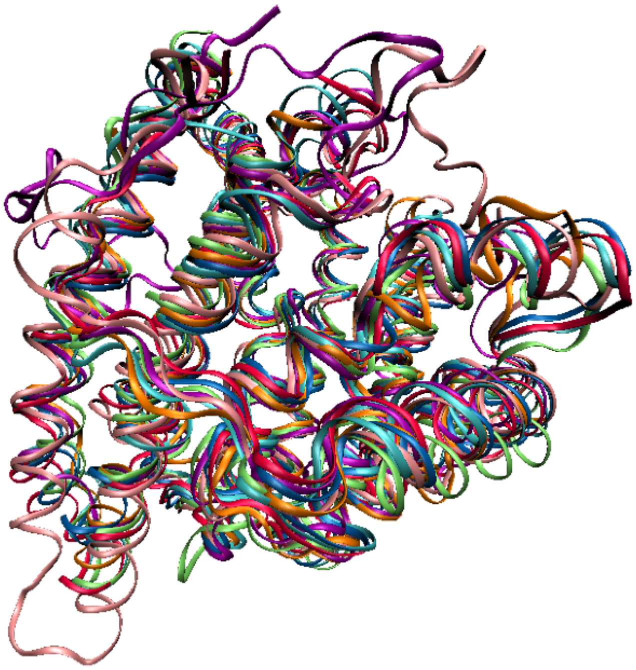

Figure S1. Overlay of several solved class I terpene synthases. ScCubS = orange, 1PS1 pentalenene synthase = teal, 3KB9 epi-isozizaene synthase = pink, 4MC3 hedycaryol synthase = lime, 4OKM selinadiene synthase = purple, 5NX5 linalool/nerolidol synthase = light blue, 5NX7 1,8-cineole synthase= red.

R202, along with T205 and S206 (residue numbering as per ScCubS) forms part of a previously identified cluster responsible for substrate binding. R202 acts as a ‘PPi sensor’, and is connected to the so-called ‘effector residue’ S206 via the ‘linker residue’ T205. When GPP or FPP are sequestered from bulk solution, the R202/T205/S206 cluster is postulated to bring about an induced-fit mechanism by moving inwards and donating electron density into the C2,3 π^* molecular orbital of FPP, triggering ionisation.[13] The distance observed between C3 of the substrate and the carbonyl of S206 in our docking models suggest the structure is in an open/partially open conformation.

Mechanism of 10-*epi*-cubebol formation

Making the cubebane scaffold, from which 10-*epi*-cubebol is derived, from FPP requires multiple highly selective steps and some of Nature's most difficult chemistry. FPP must first be ionised to the farnesyl cation, and then isomerised to the nerolidyl cation (via nerolidyl pyrophosphate (NPP)), giving the correct conformation for initial cyclisation. Failure to isomerise most likely accounts for the presence of (*E*)-β-farnesene, which, along with the hydroxylated farnesol, may result directly from enzyme action, or else from the action of endogenous FPP-processing enzymes in *E. coli.*[16] Cation A is the result of the first cyclisation step. From here, premature quenching must be blocked, and sufficient stabilisation offered to form cation B by a 1,3 hydride shift. Cations A and B, if quenched, give germacranes, characterised by a monocyclic hydrocarbon scaffold. Although stabilisation of the allylic C1,2,3 system is required, it must not prevent the further formation of cation C via intramolecular cyclisation of cation B. This can proceed from either 'above' or 'below' the double bond at C6,7, relative to the position of the hydrocarbon tail which points above with (*R*)-stereochemistry at C10. Formation of 10-*epi*-cubebol proceeds via attack from above; attack from below eventually yields cubebol and cubenol. This step requires the stabilisation of positive charge at C7. A 1,2 hydride shift is then required to displace the positive charge from C7 (cation C) to C6 (cation D), giving the correct stereochemistry for the third and final cyclisation step. Premature quenching of cations C and D gives the cadalanes, characterised by a bicyclic hydrocarbon scaffold. An example is 1,10-di-*epi*-cubenol, which is achieved by hydroxylation of cation D. Finally, the third intramolecular cyclisation of cation D gives the tricyclic cubebane scaffold (cation E). The resulting positive charge on C3 is quenched by water to deliver the main product, 10-*epi*-cubebol.

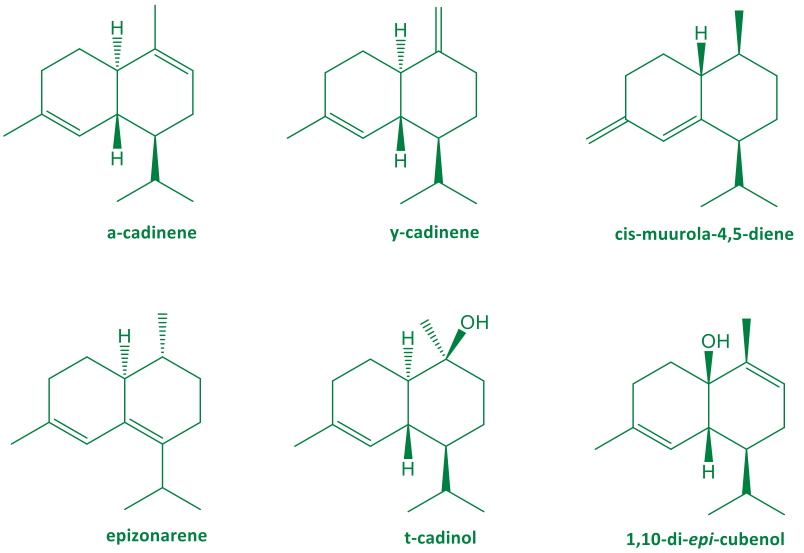

Figure S2. Select cadalane compounds produced by the S206G and F211A variants. These compounds are characterised by a bicyclic hydrocarbon scaffold, and result from the quenching of cations C and D.

Choosing the conformation of FPP

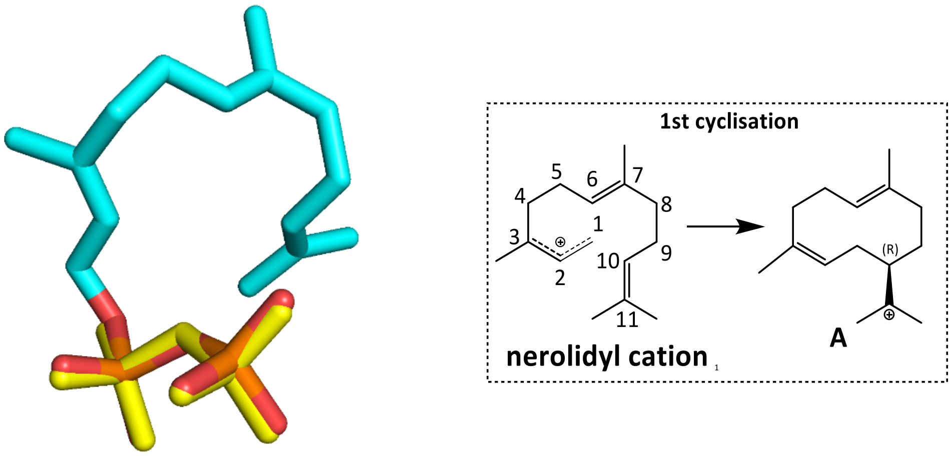

Figure S3. Selecting the best FPP docking conformer. The PPi (red) of the best FPP docking conformer is closely aligned with the PPi as bound in the ScCubS-$Mg^{2+}{}_3$-PPi crystal structure (yellow). The docking pose has the appropriate conformation for the first cyclisation (dashed box, right). It is also positioned correctly with respect to the effector triad (Figure 4).

DFT calculations and docking of intermediates

We modelled cations B and C, as well as germacradien-4-ol, using DFT and the docked FPP structure. Additional experimental and structural data are required to fully validate these models, but these geometry optimised structures adopt catalytically relevant conformations in the active site when aligned with FPP, consistent with our proposed FPP docking pose and the relevant contacts between our mutated amino acid residues (Figure S4).

Furthermore, we docked the geometry optimised intermediates into ScCubS. The top 8 conformers were ordered according to AutoDock's binding score, and in all three cases the docking conformer shown in Figure S4 was within 1.8 kcal/mol of the top result, which itself could be disregarded as obviously incorrect (e.g. upside down, clashing with the PPi in our crystal structure, etc.).

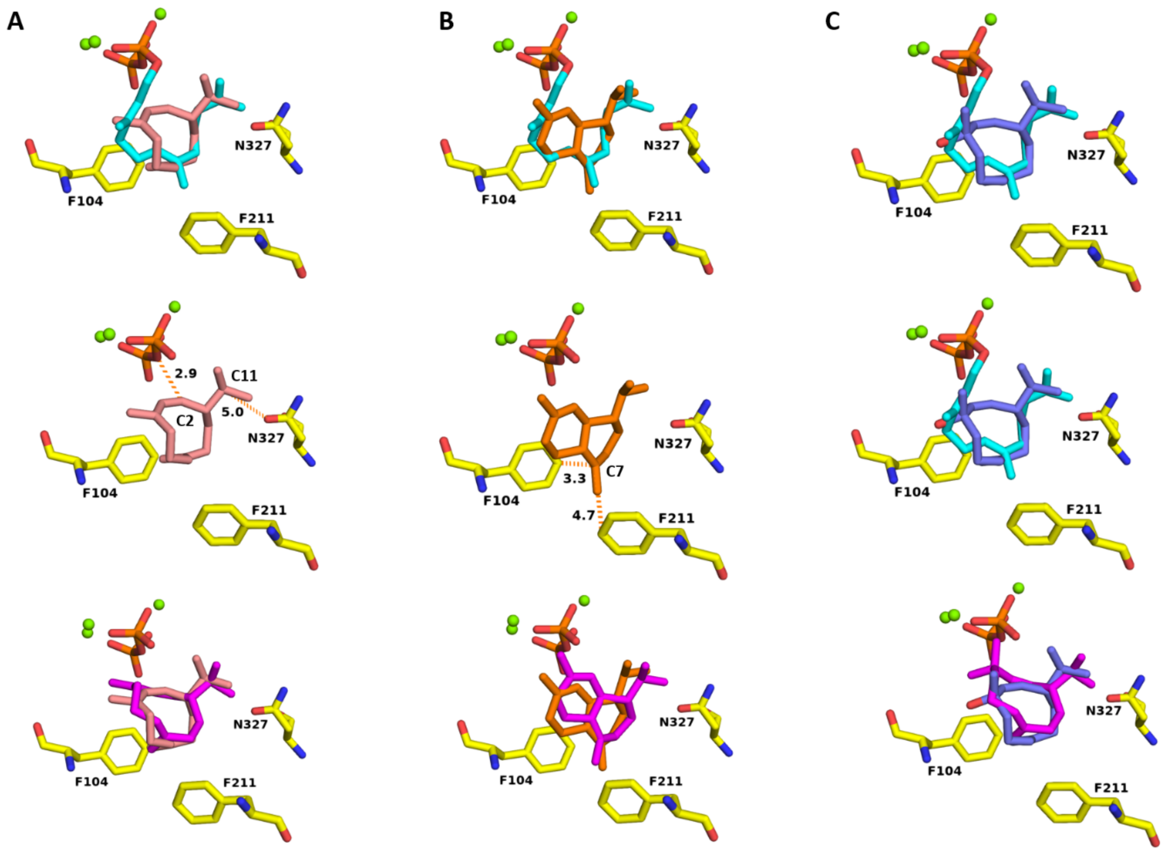

Figure S4. DFT geometry optimised intermediates and docking. A) Top: geometry optimised cation B (pink) aligned with FPP (blue); middle: distances to key residues; bottom: cation B docking result (magenta). Positive charge exists on C2 after a 1,3 hydride shift from C11 (cation A). We assume that the PPi remains in a similar position to the docked FPP. B) Top: geometry optimised cation C (orange) alignment with FPP; middle: distances to key residues; bottom: cation C docking result (magenta). Positive charge exists on C7 after ring closure. C) Top: geometry optimised germacradien-4-ol (dark blue) aligned with FPP; middle: germacradien-4-ol is fully quenched; bottom: germacradien-4-ol docking result (magenta).

We postulate that: N327 adds negative charge in the active site region surrounding C11, C1, C2. There is likely additional negative charge in the region of C1, C2, C11 if the abstracted PPi remains in a similar position to that of the docked FPP (we assume it does). F104 and F211 can stabilise positive charge near C7 by cation-π interactions. These regions are where the intermediates are expected to carry positive charge according to our mechanism in Figure 1. There may be kinetic competition between ring closure to form Cation C and water attack to form germacradien-4-ol. Additional charge from N327D (or lack of cation-π interactions in

F104A) could be sufficient to stabilise Cation B long enough for hydroxylation to outcompete ring closure, consistent with our mutagenesis data.

Sequence alignment and the common effector residues in bacterial terpene synthases

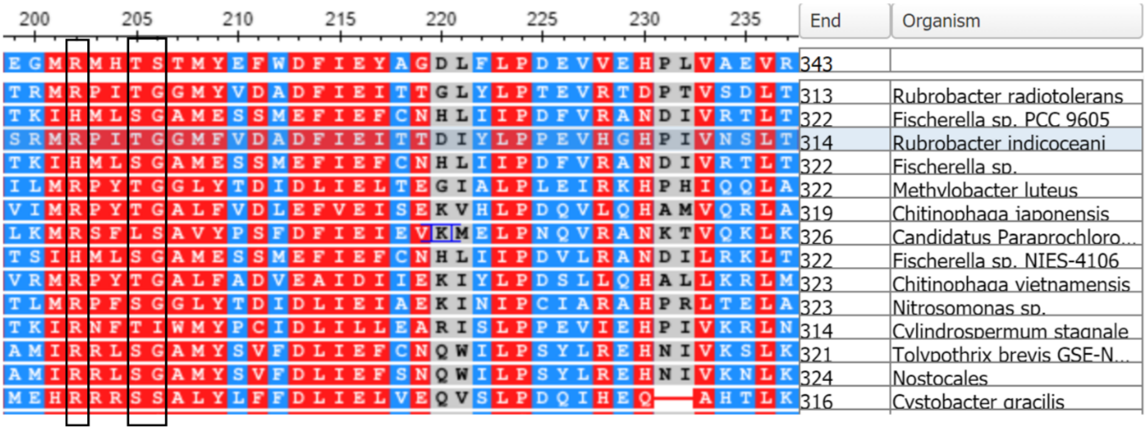

Figure S5. Sequence alignment of non-redundant bacterial protein sequences with the highest sequence identity to ScCubS. The primary amino acid sequence of ScCubS was queried against non-redundant protein sequences with the organism defined as bacteria using the 'blastp' (protein-protein BLAST) algorithm. [15] The sensor-linker-effector triad is highlighted with black boxes. Sample from a comparison of 100 sequences.

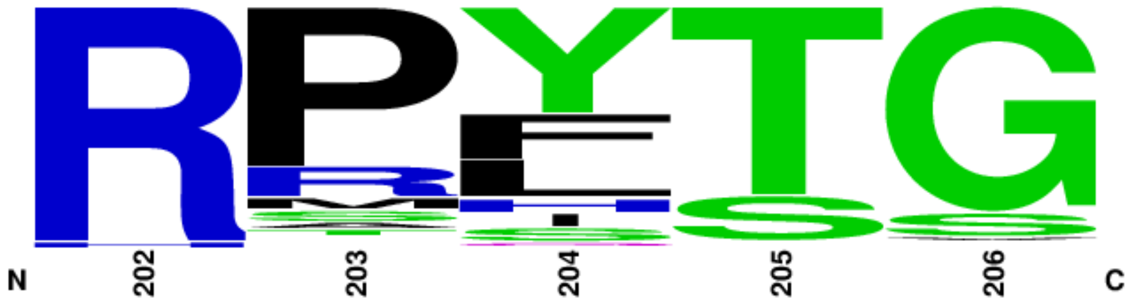

Figure S6. Sequence logo showing amino acid frequency for the sensor-linker-effector triad. The frequencies shown here are determined by comparison of the 100 non-redundant bacterial protein sequences with highest sequence identity to ScCubS. The PPi sensor arginine (202) is totally conserved (the small number of variations are most likely inactive). The linker (205) is most commonly threonine,

as observed in ScCubS. The effector (206) is overwhelmingly glycine. The frequency logo was generated from the results of the alignment described in Figure S4 and employs the following colour scheme: positively charged = blue; non-polar = black; polar (and glycine) = green.

GC and GCMS analysis

Chemicals and standards

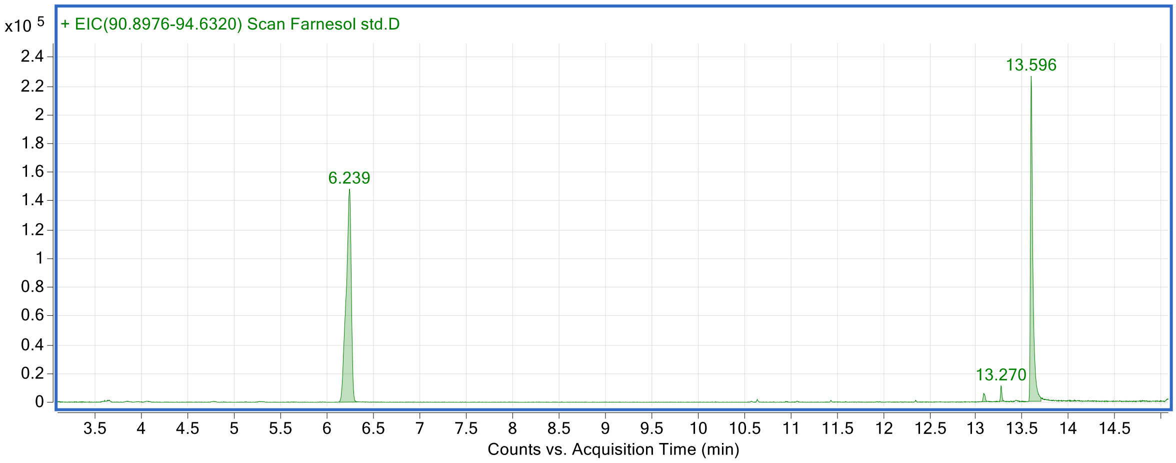

Figure S7: Chromatogram for authentic standard farnesol. 6.239 = internal standard; 13.270 = farnesal; 13.596 = farnesol.

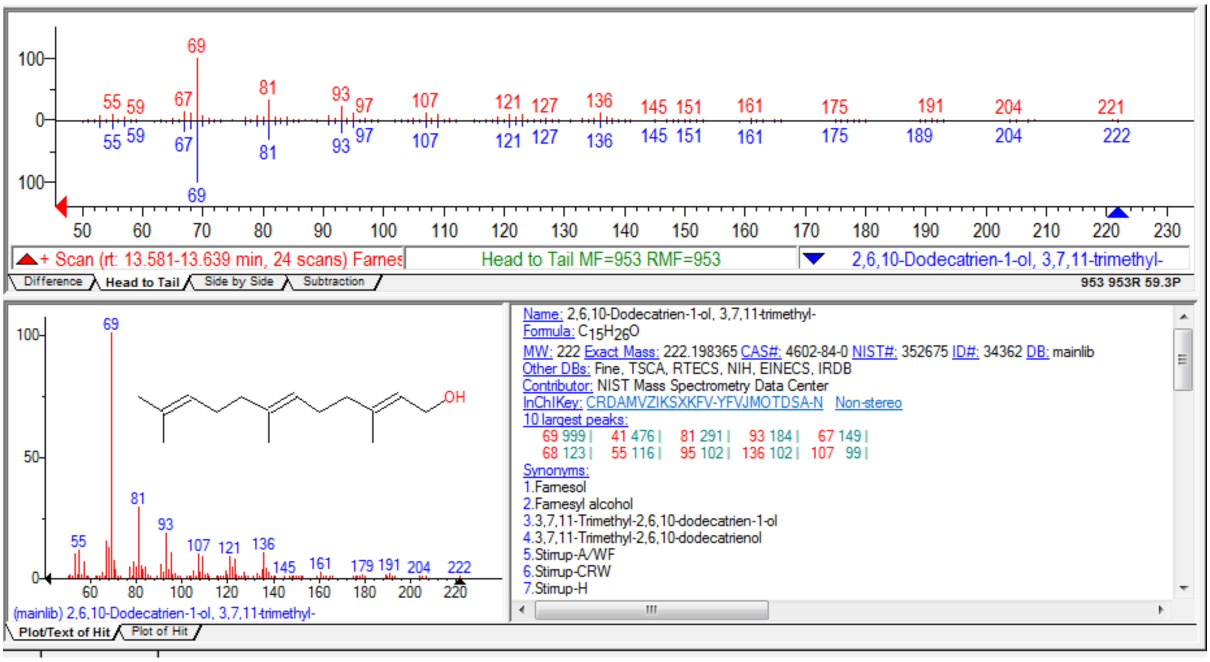

Figure S8: Mass spectra comparison of authentic standard farnesol against NIST database.

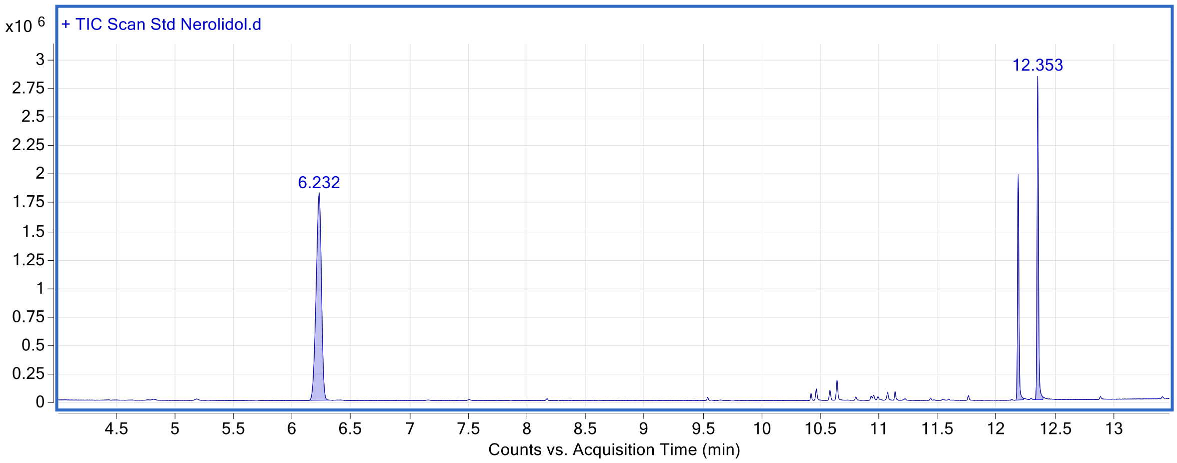

Figure S9: Chromatogram for authentic standard nerolidol. 6.232 = internal standard; 12.186 = *cis*-nerolidol; 12.353 = *trans*-nerolidol

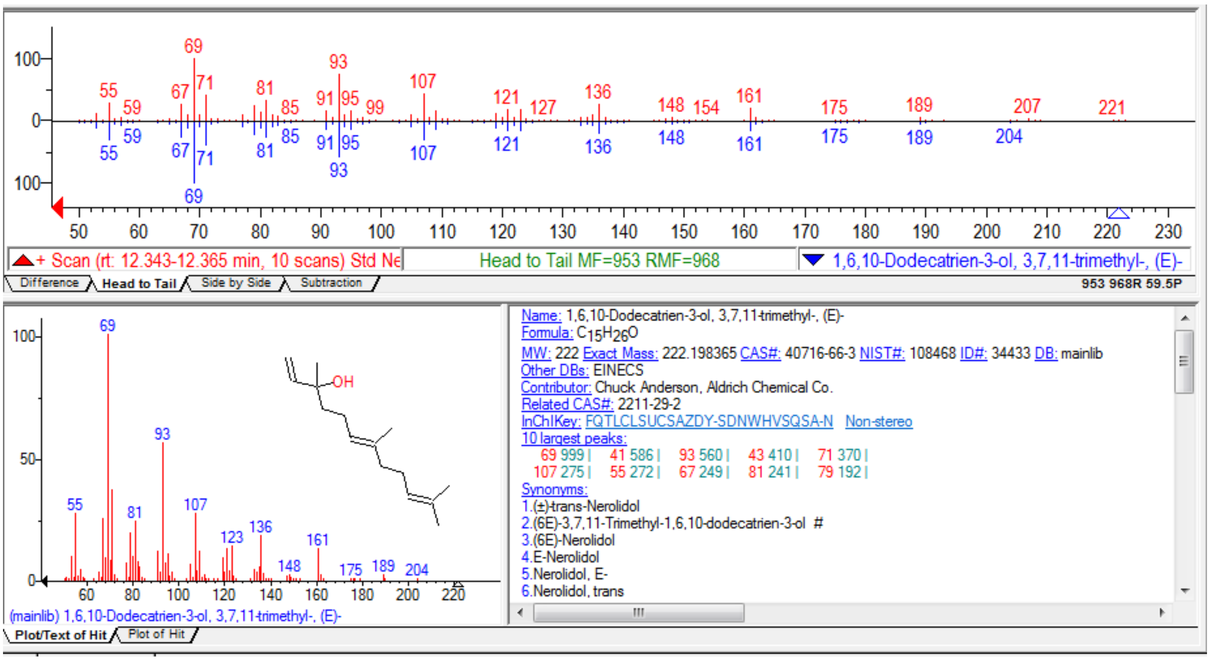

Figure S10: Mass spectra comparison of authentic standard nerolidol against NIST database.

Mass spectra for identified sesquiterpenes

In the absence of authentic standards, sesquiterpene products were identified using MS hits in the NIST database, their retention indices[19], and comparison of order of elution with the previously-determined WT product profile of ScCubS[1].

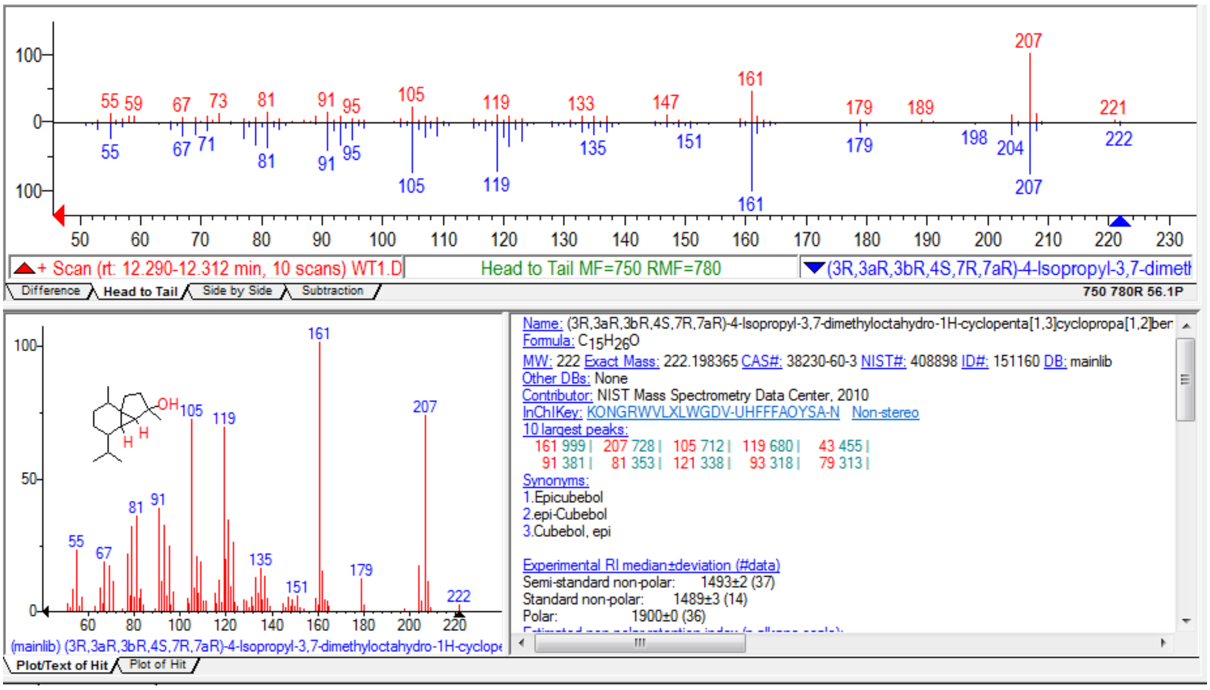

Figure S11: Mass spectra comparison of sesquiterpene identified as 10-*epi*-cubebol (sample = red, reference compound = blue).

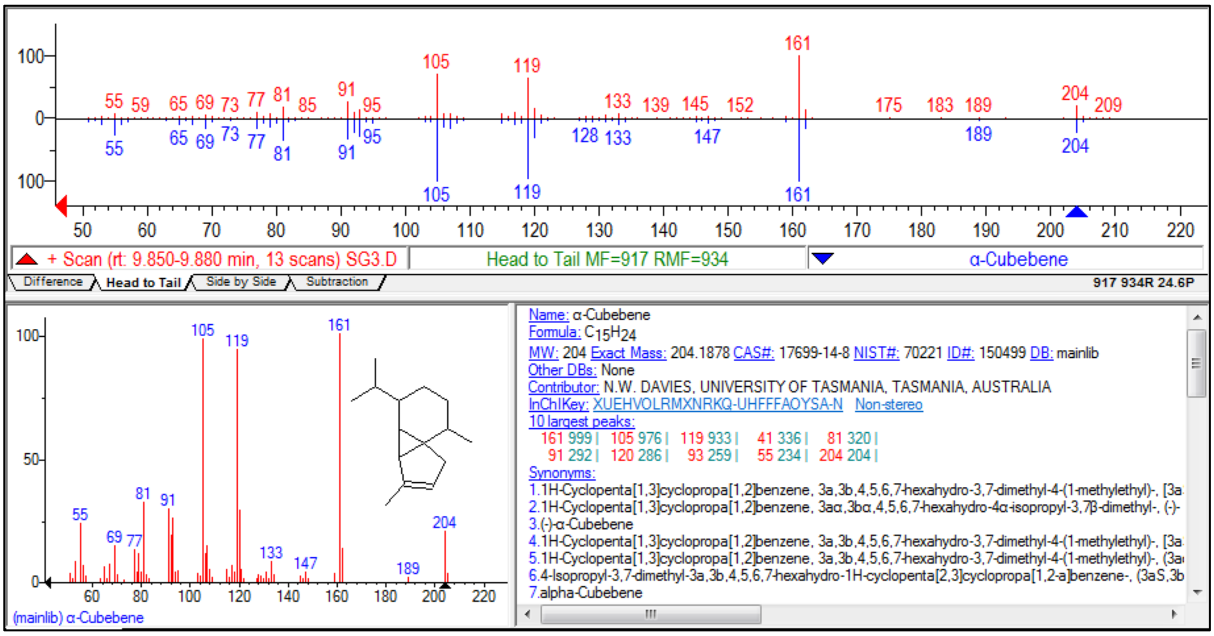

Figure S12: Mass spectra comparison of sesquiterpene identified as α-cubebene (sample = red, reference compound = blue).

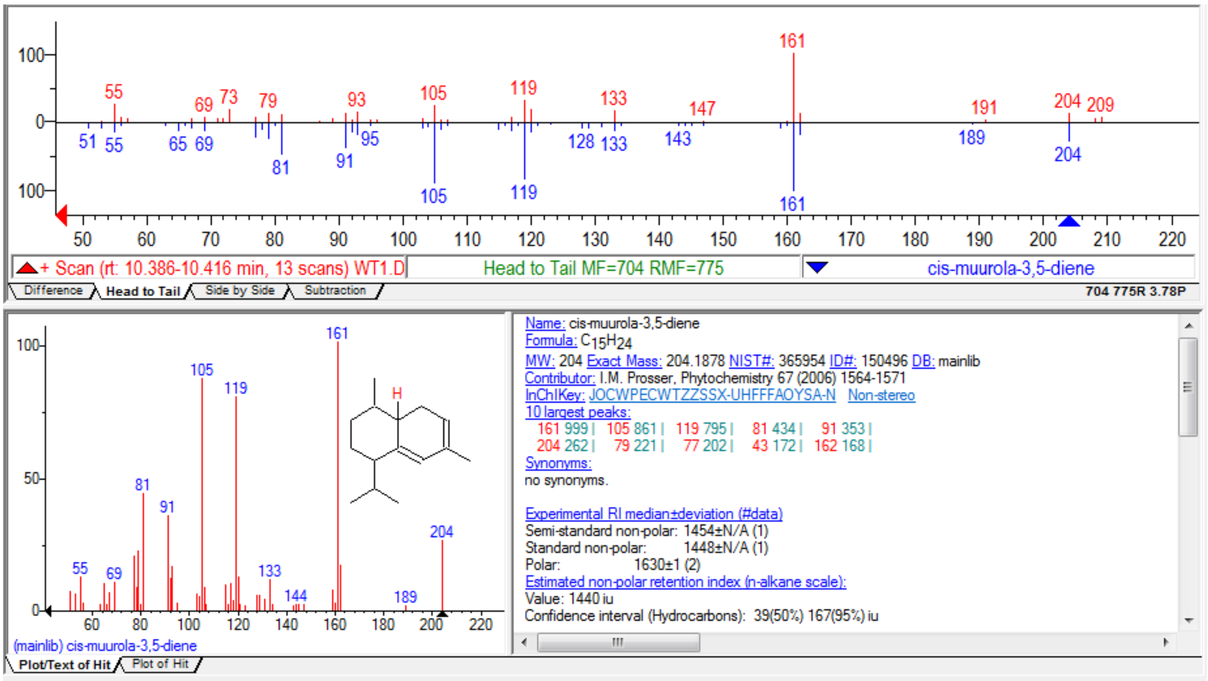

Figure S13: Mass spectra comparison of sesquiterpene identified as cis-muurola-3,5-diene (sample = red, reference compound = blue).

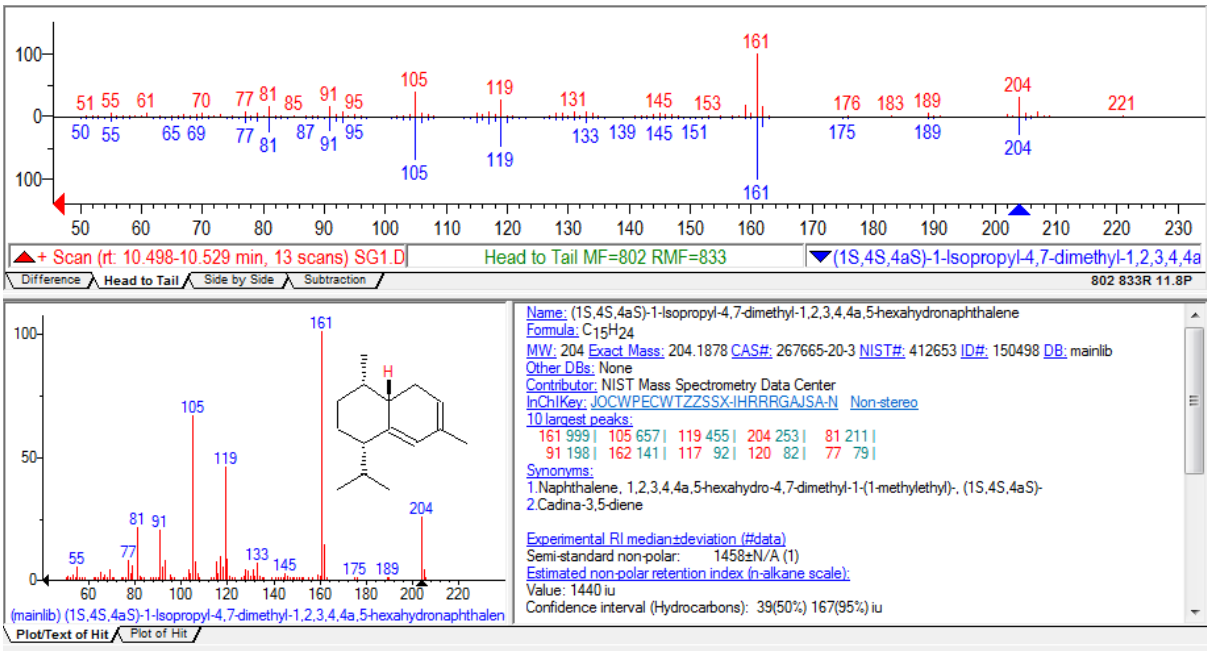

Figure S14: Mass spectra comparison of sesquiterpene identified as cadina-3,5-diene (sample = red, reference compound = blue).

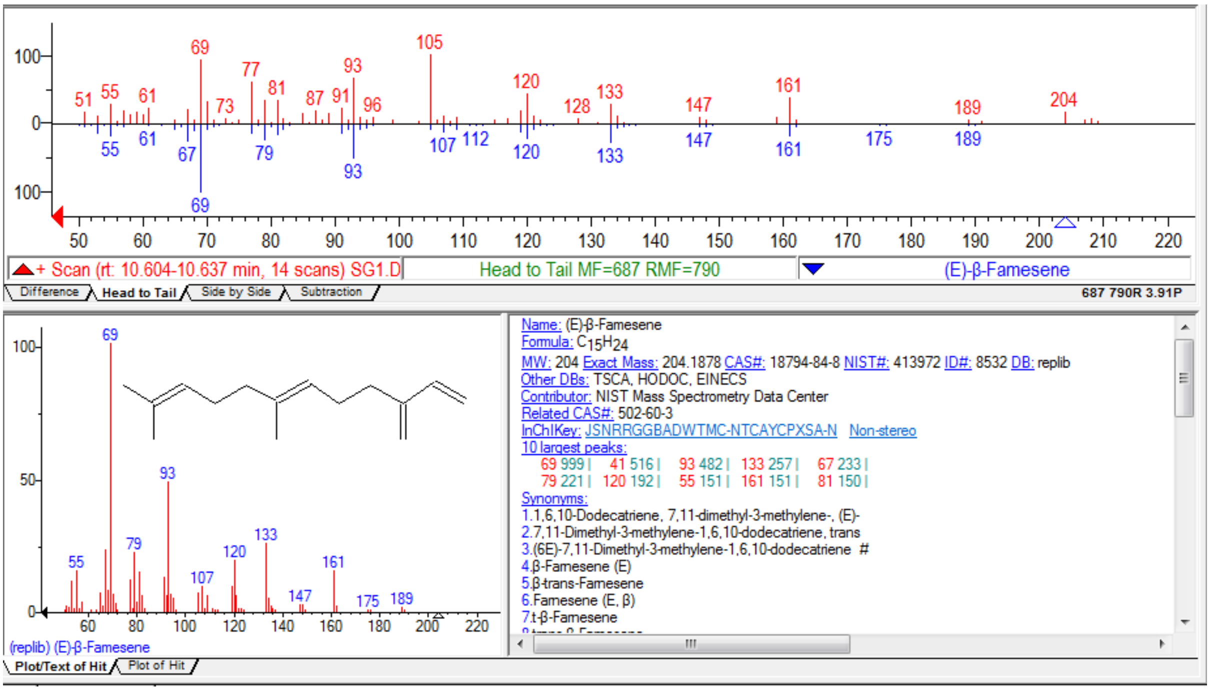

Figure S15: Mass spectra comparison of sesquiterpene identified as (E)-β-farnesene (sample = red, reference compound = blue).

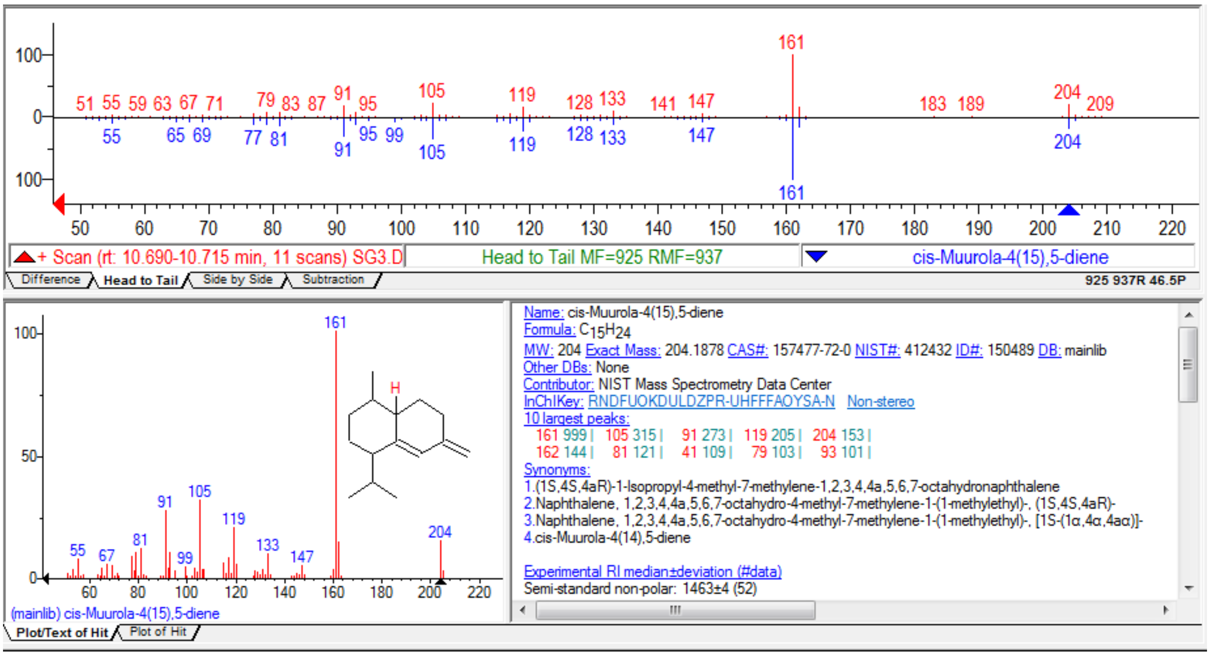

Figure S16: Mass spectra comparison of sesquiterpene identified as cis-muurola-4,5-diene (sample = red, reference compound = blue).

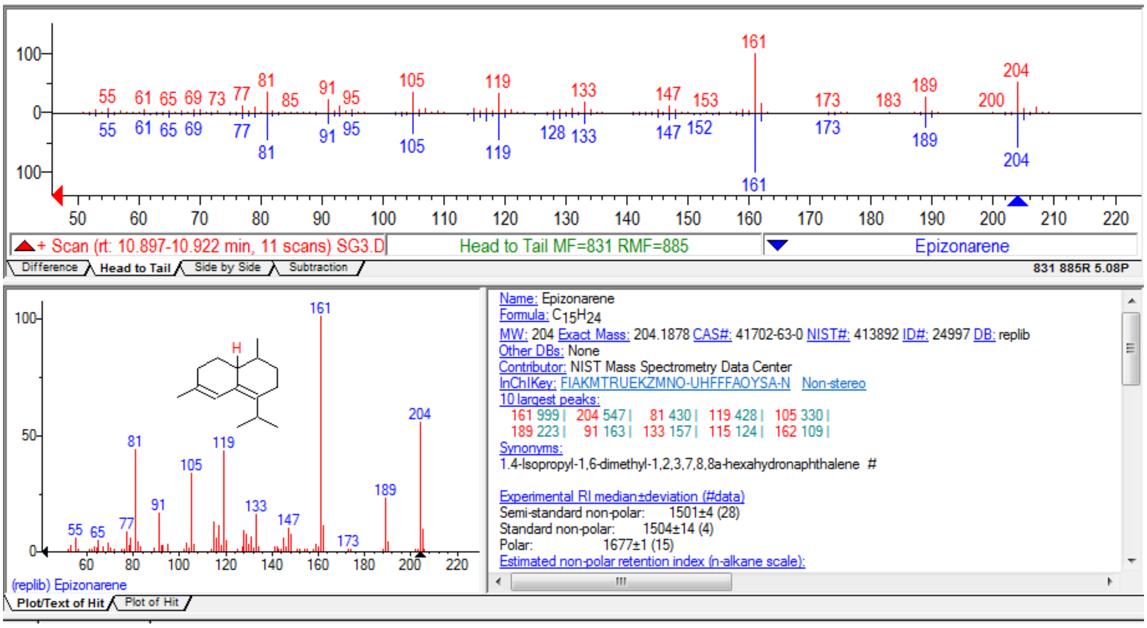

Figure S17: Mass spectra comparison of sesquiterpene identified as epizonarene (sample = red, reference compound = blue).

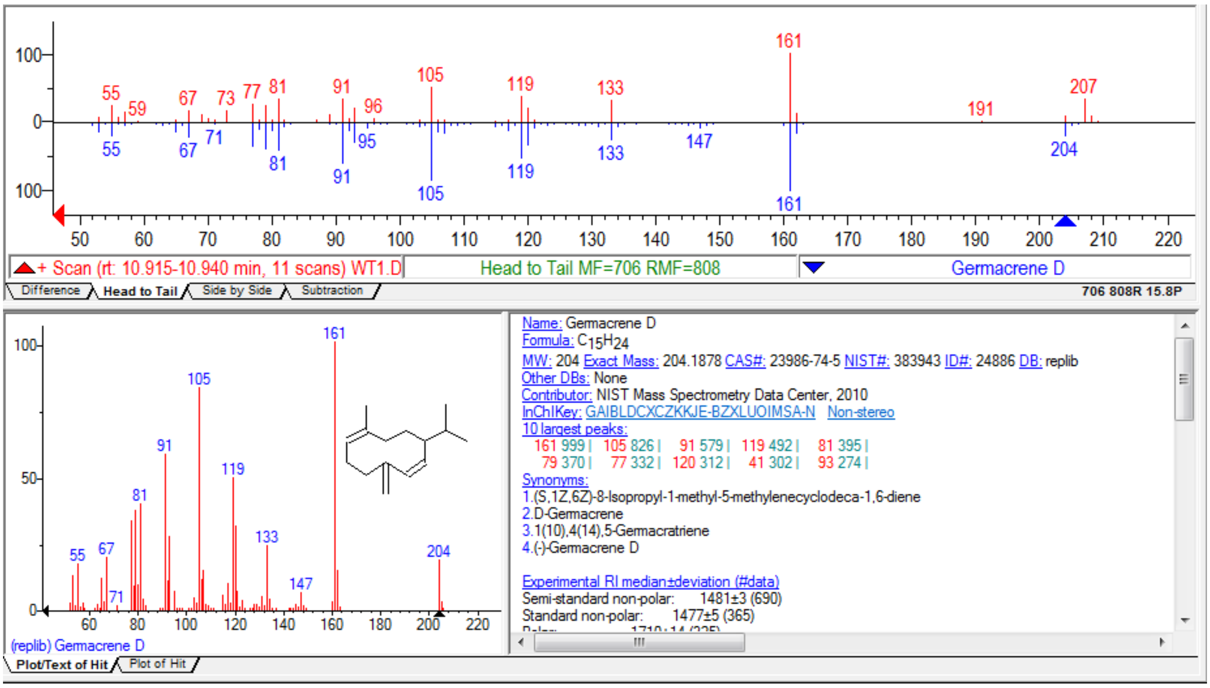

Figure S18: Mass spectra comparison of sesquiterpene identified as germacrene D (sample = red, reference compound = blue).

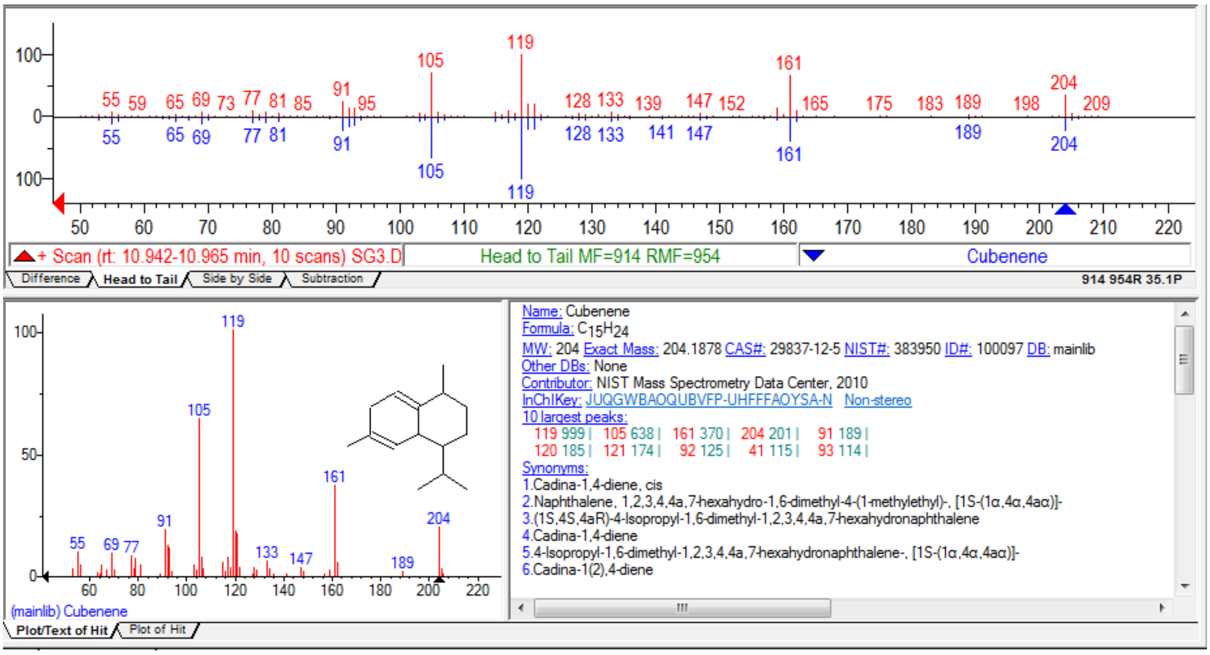

Figure S19: Mass spectra comparison of sesquiterpene identified as cadina-1,4-diene (sample = red, reference compound = blue).

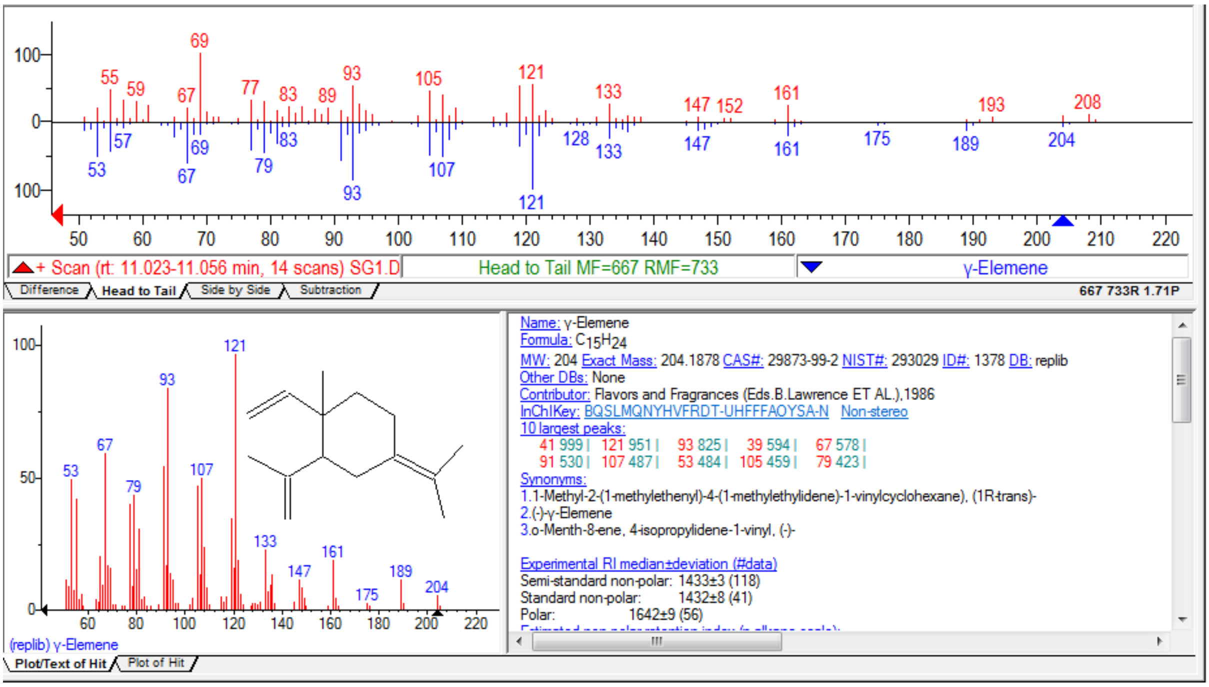

Figure S20: Mass spectra comparison of sesquiterpene identified as γ-elemene (sample = red, reference compound = blue).

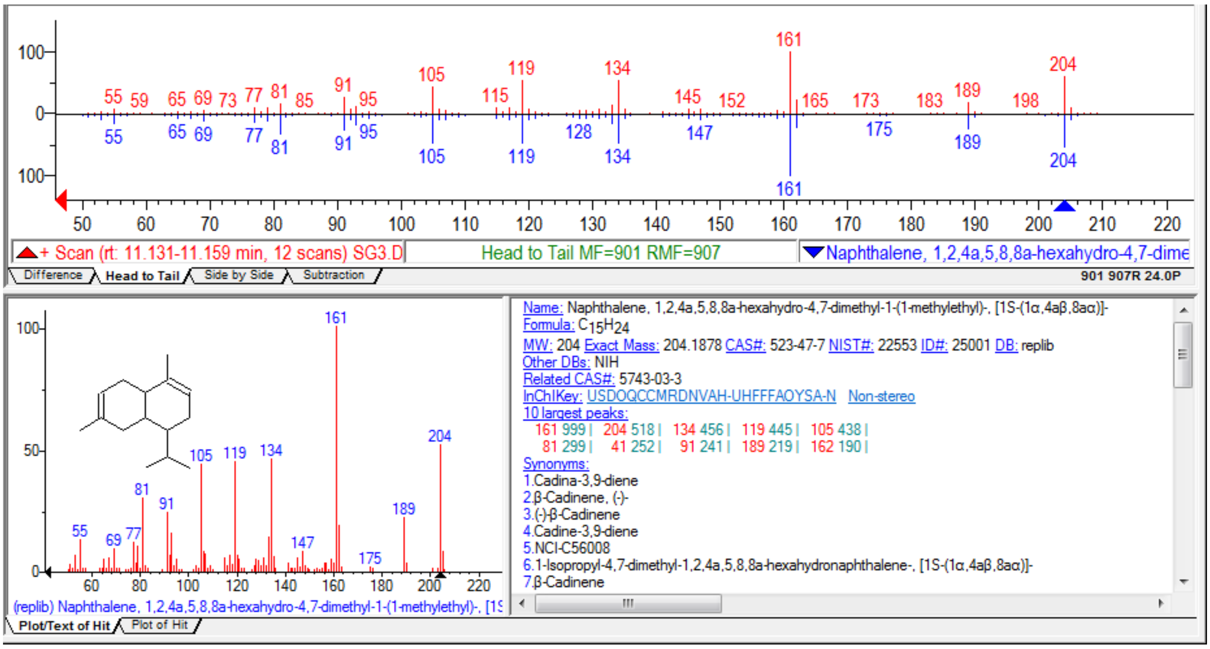

Figure S21: Mass spectra comparison of sesquiterpene identified as cadina-3,9-diene (sample = red, reference compound = blue).

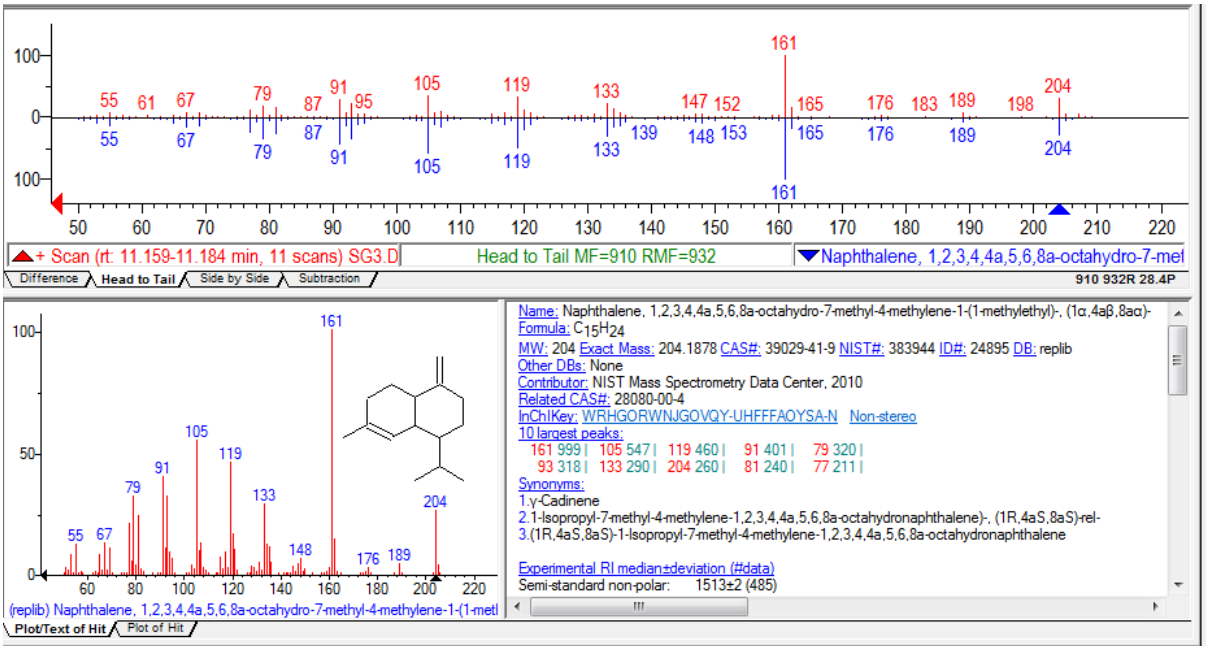

Figure S22: Mass spectra comparison of sesquiterpene identified as γ-cadinene (sample = red, reference compound = blue).

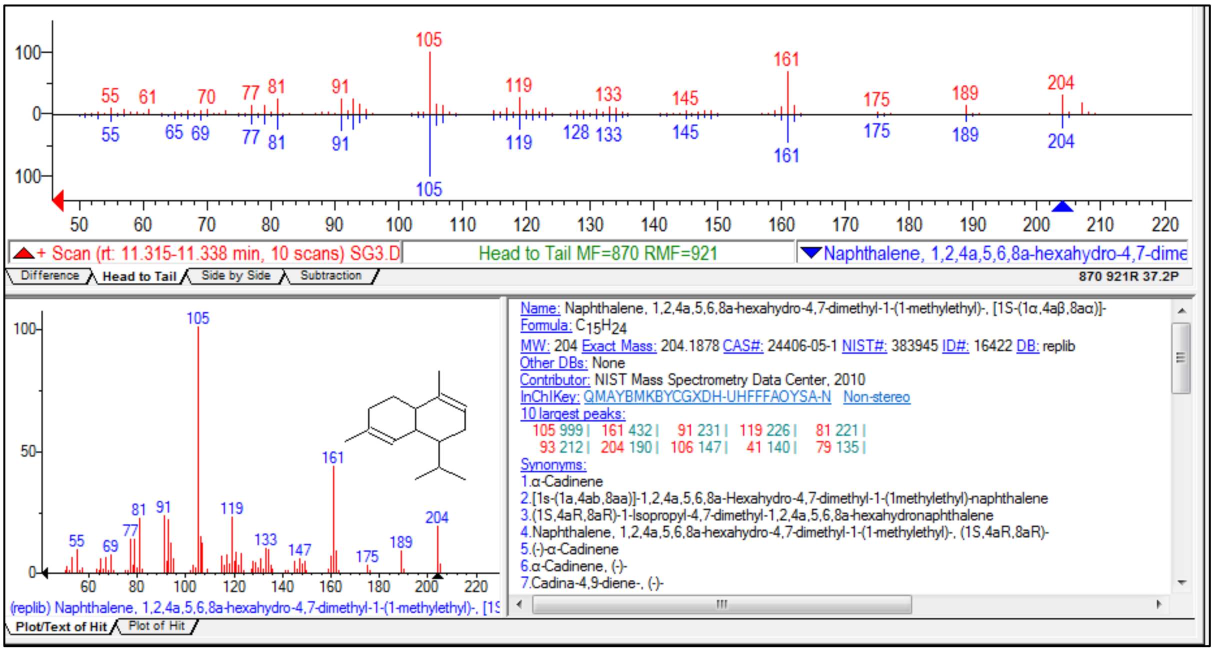

Figure S23: Mass spectra comparison of sesquiterpene identified as α-cadinene (sample = red, reference compound = blue).

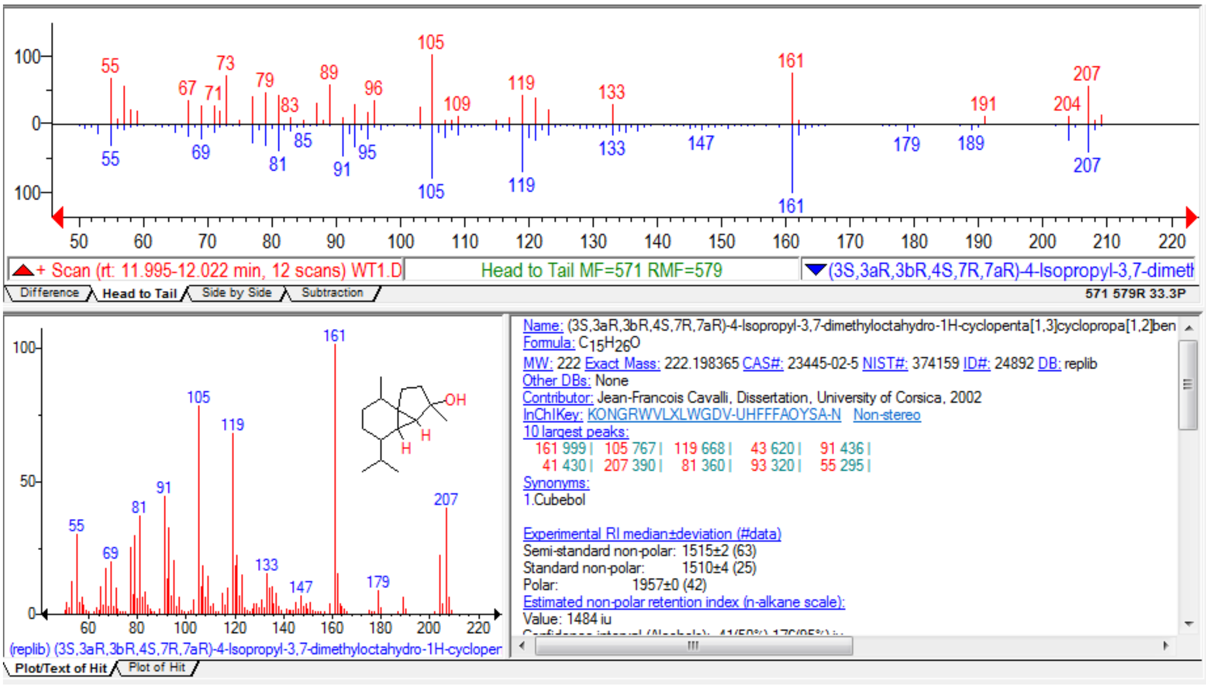

Figure S24: Mass spectra comparison of sesquiterpene identified as cubebol (sample = red, reference compound = blue).

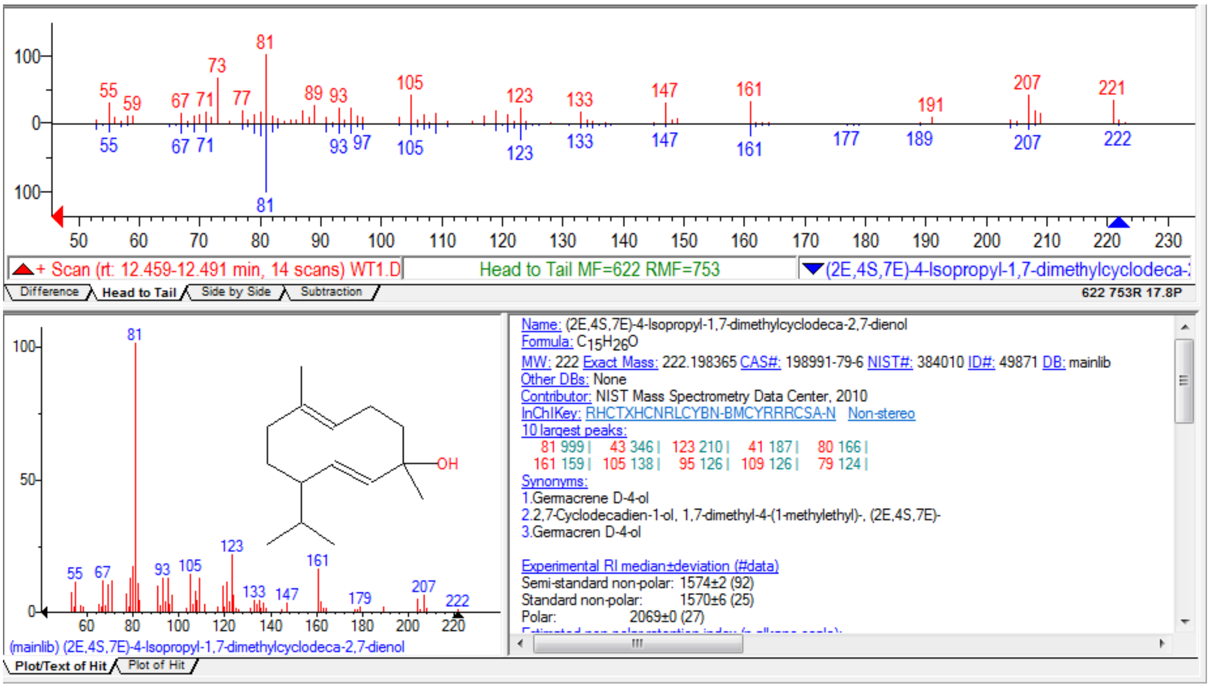

Figure S25: Mass spectra comparison of sesquiterpene identified as germacradien-4-ol (sample = red, reference compound = blue).

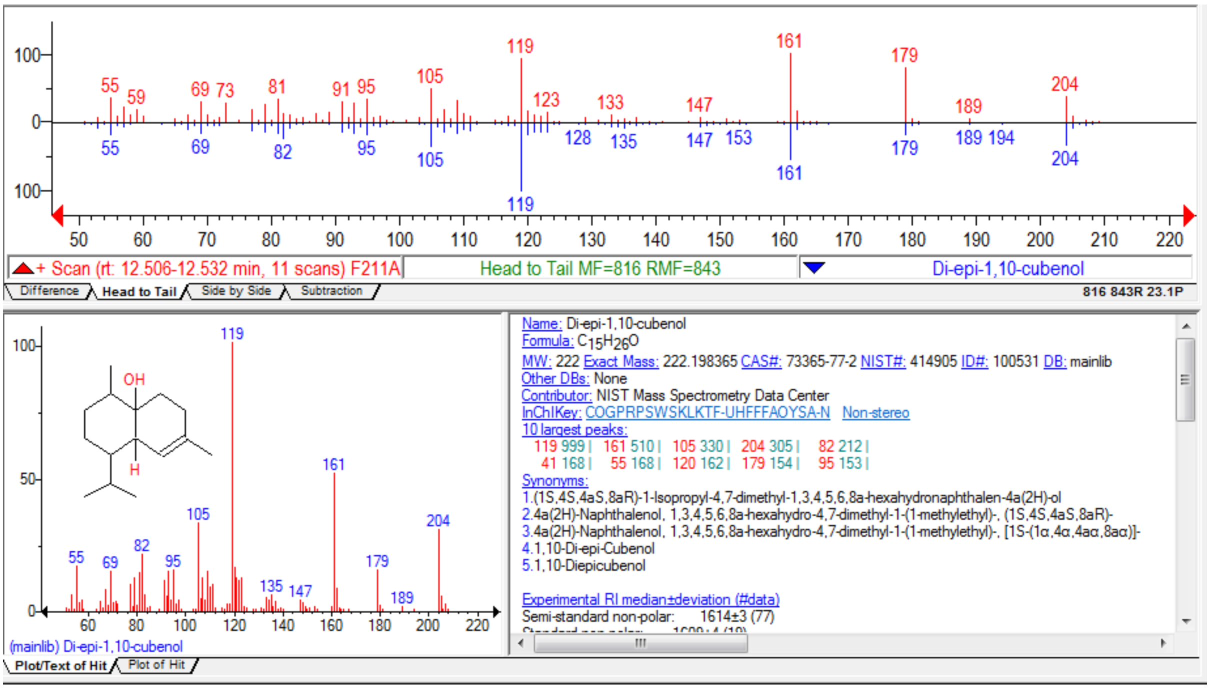

Figure S26: Mass spectra comparison of sesquiterpene identified as 1,10-di-*epi*-cubenol (sample = red, reference compound = blue).

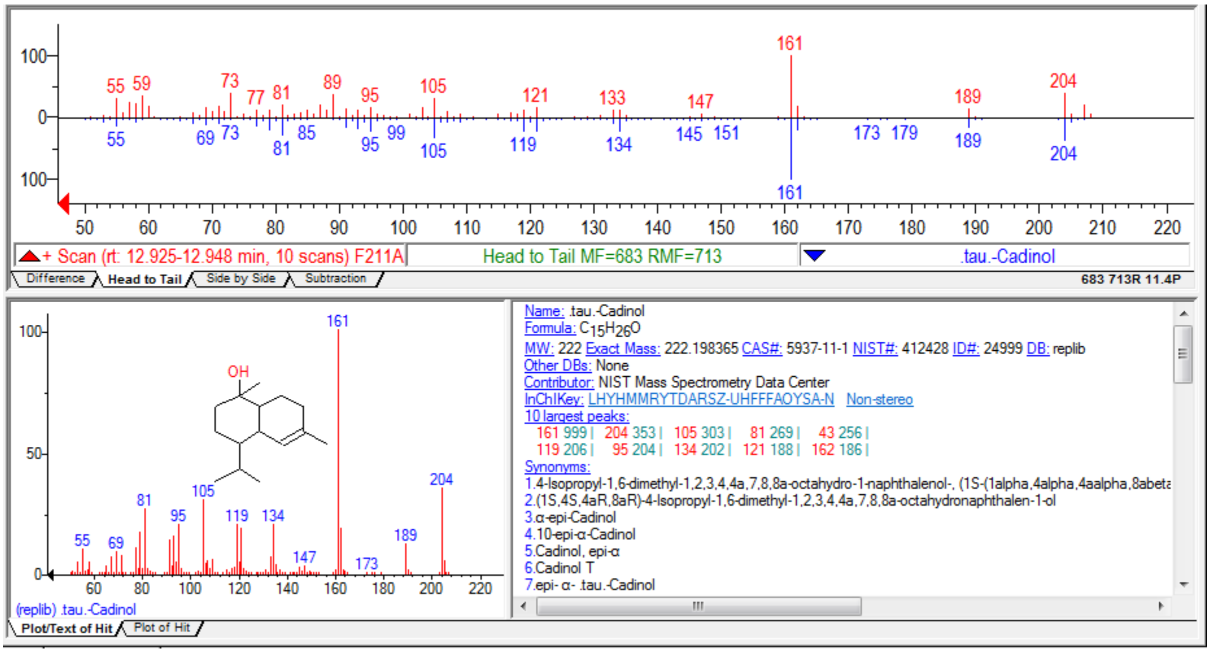

Figure S27: Mass spectra comparison of sesquiterpene identified as τ-cadinol (sample = red, reference compound = blue).

GCMS chromatograms for variants

The sesquiterpenoids made by ScCubS and other TSs show a characteristic peak at m/z = 161. Extracted ion count (EIC) spectra at m/z = 161 are shown below, and represent the sesquiterpenoid product profile of each variant. Monoterpenoids show a characteristic peak at m/z = 93, but these peaks are not shown here as monoterpenes are not the predominant focus of this work (GPP is not a natural substrate for ScCubS).

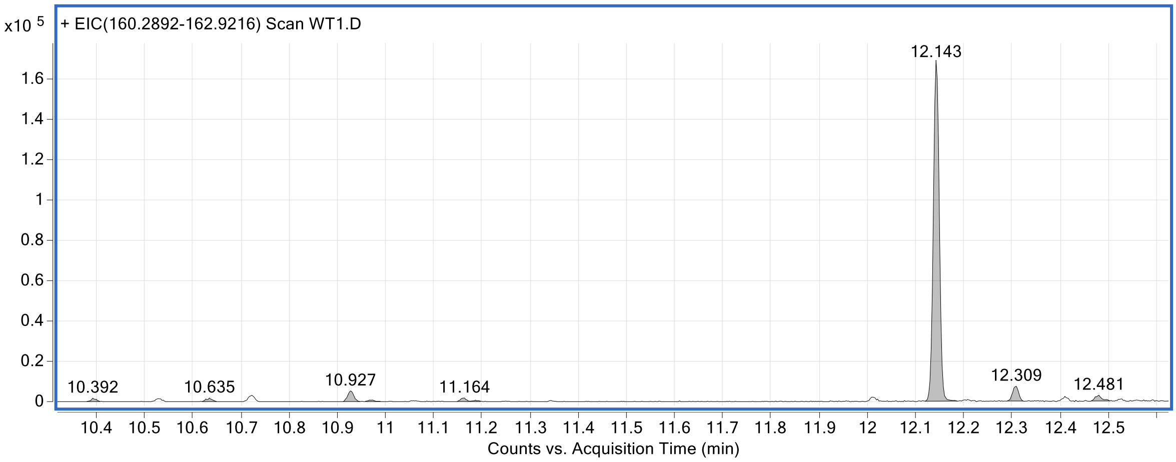

Figure S28: Extracted ion chromatogram (EIC) at m/z = 161 for ScCubS WT. 10.392 = cis-muurola-4,5-diene; 10.635 = (*E*)-β-farnesene; 10.927 = germacrene D; 11.164 = γ-cadinene; 12.143 = 10-*epi*-cubebol; 12.309 = cubebol; 12.481 = germacradien-4-ol.

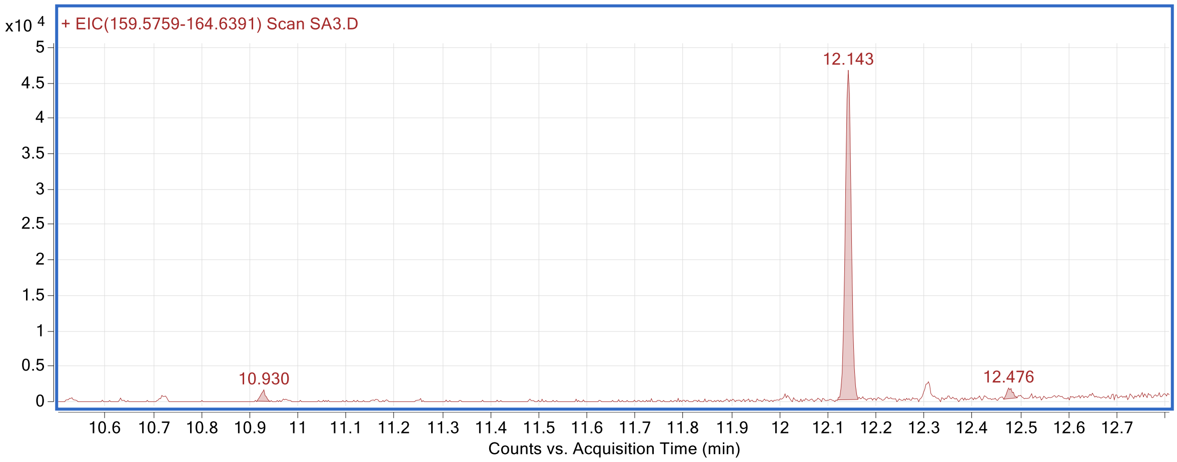

Figure S29: Extracted ion chromatogram (EIC) at m/z = 161 for S206A. 10.930 = germacrene D; 12.143 = 10-*epi*-cubebol; 12.476 = germacradien-4-ol.

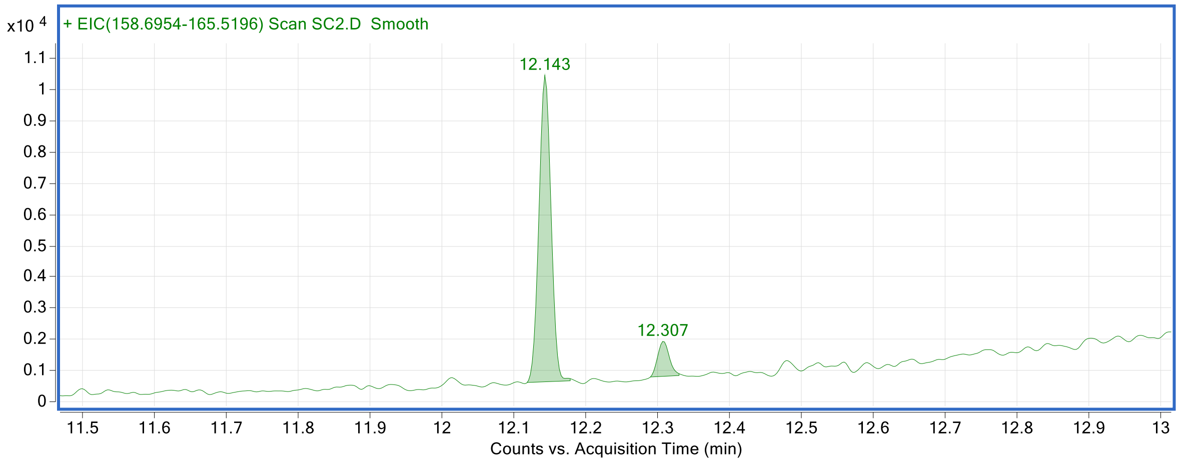

Figure S30: Extracted ion chromatogram (EIC) at m/z = 161 for S206C. 12.143 = 10-*epi*-cubebol; 12.307 = cubebol.

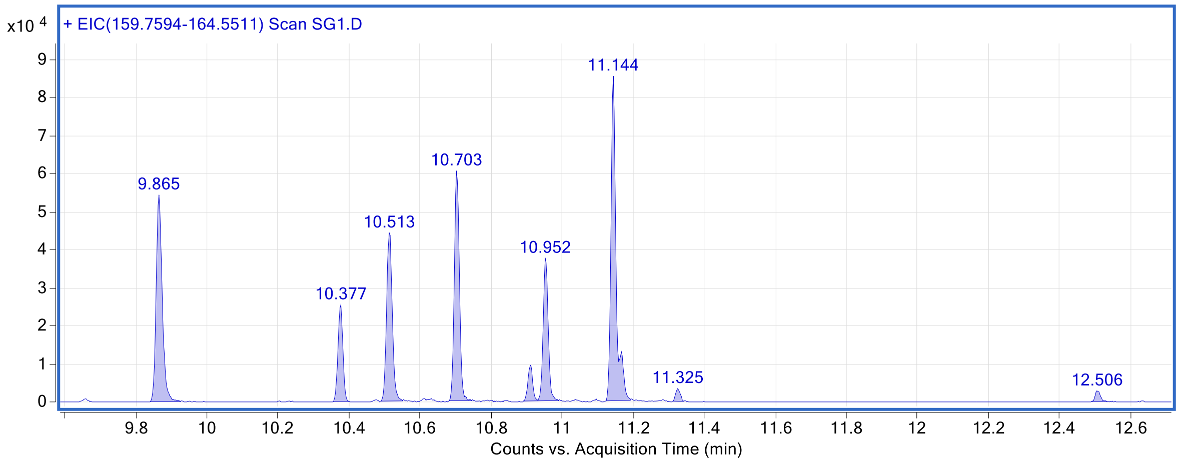

Figure S31: Extracted ion chromatogram (EIC) at m/z = 161 for S206G. 9.865 = α-cubebene; 10.377 = cis-muurola-3,5-diene; 10.513 = cadina-3,5-diene; 10.703 = cis-muurola-4,5-diene; 10.909 = epizonarene; 10.952 = cadina-1,4-diene; 11.144 = cadina-3,9-diene; 11.325 = α-cadinene; 12.506 = 1,10-di-*epi*-cubenol.

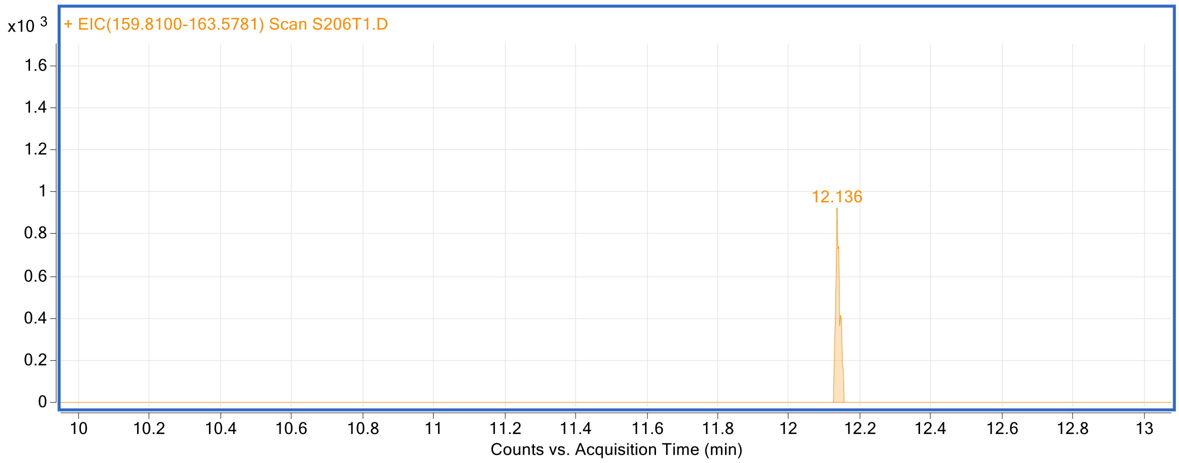

Figure S32: Extracted ion chromatogram (EIC) at m/z = 161 for S206T. 12.136 = 10-*epi*-cubebol.

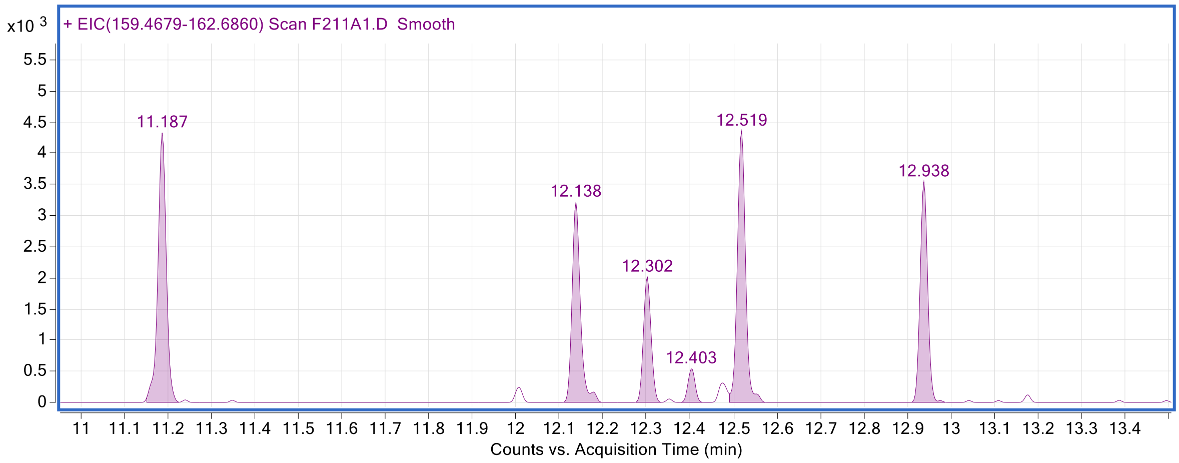

Figure S33: Extracted ion chromatogram (EIC) at m/z = 161 for F211A. 11.187 = γ-cadinene; 12.138 = 10-*epi*-cubebol; 12.302 = cubebol; 12.519 = 1,10-di-*epi*-cubenol; 12.938 = τ-cadinol.

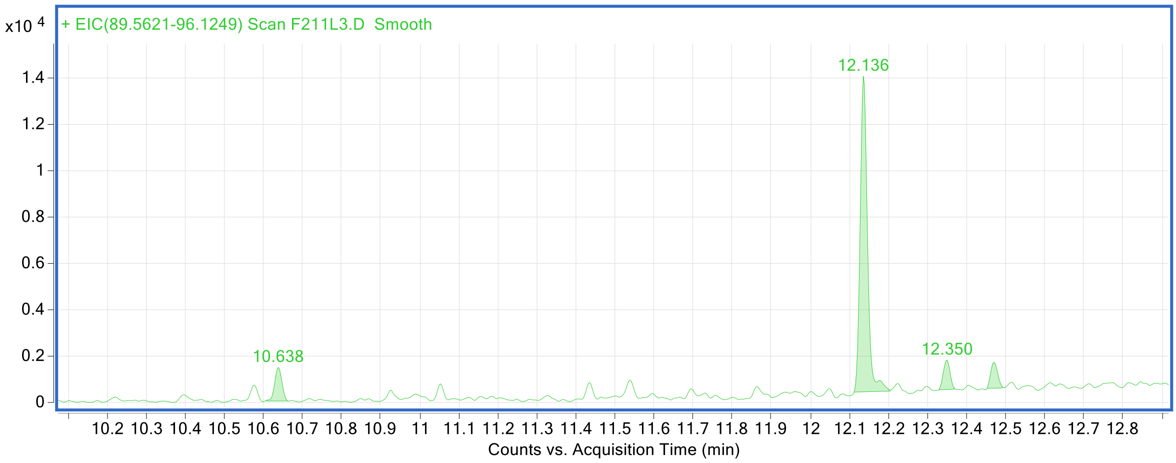

Figure S34: Extracted ion chromatogram (EIC) at m/z = 161 for F211L. 10.638 = (*E*)-β-farnesene; 10.970 = α-cubebene; 12.136 = 10-*epi*-cubebol; 12.350 = nerolidol; 12.470 = germacradien-4-ol.

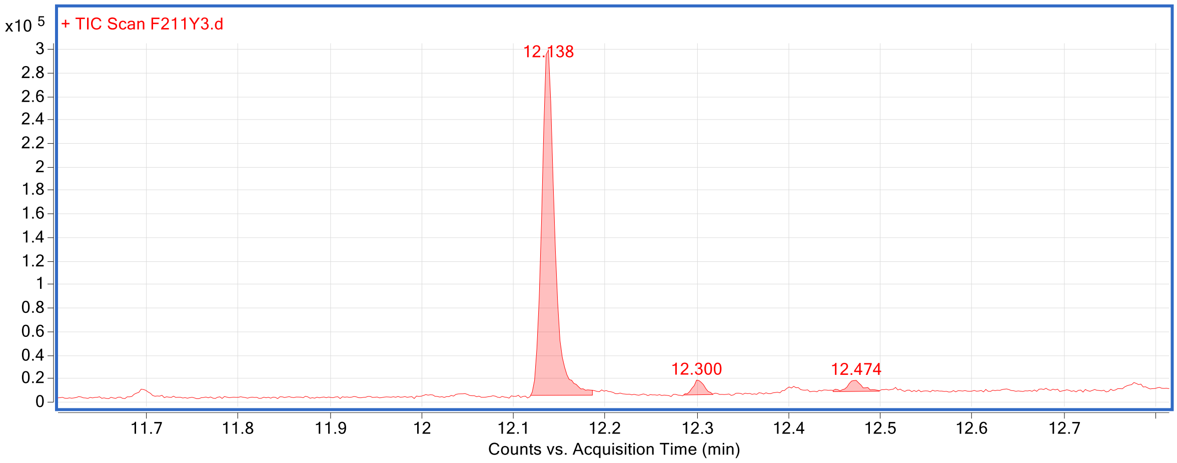

Figure S35: Extracted ion chromatogram (EIC) at m/z = 161 for F211Y. 12.138 = 10-*epi*-cubebol; 12.300 = cubebol; 12.474 = germacradien-4-ol.

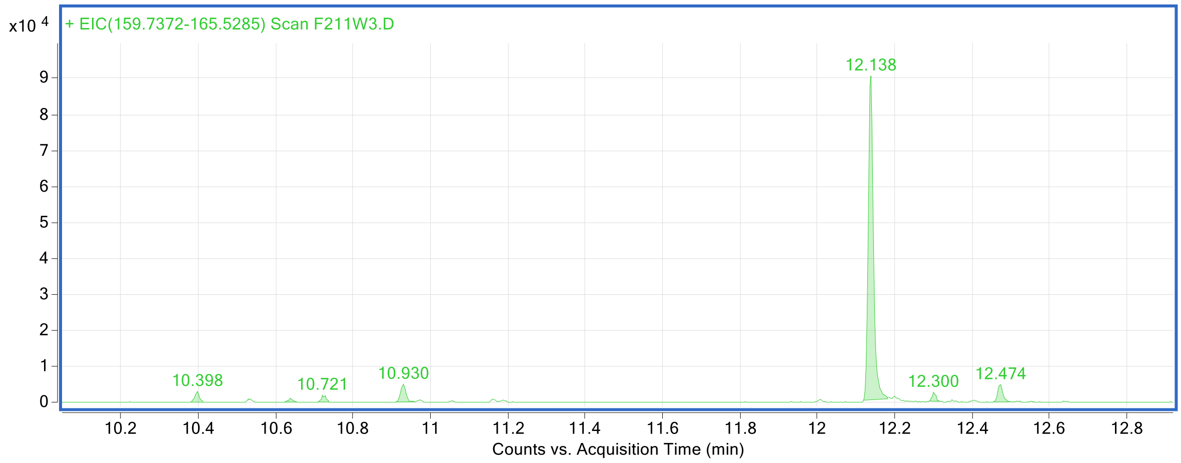

Figure S36: Extracted ion chromatogram (EIC) at m/z = 161 for F211W. 10.398 = cis-muurola-4,5-diene; 10.638 = (*E*)-β-farnene; 10.930 = germacrene D; 12.138 = 10-*epi*-cubebol; 12.300 = cubebol; 12.474 = germacradien-4-ol.

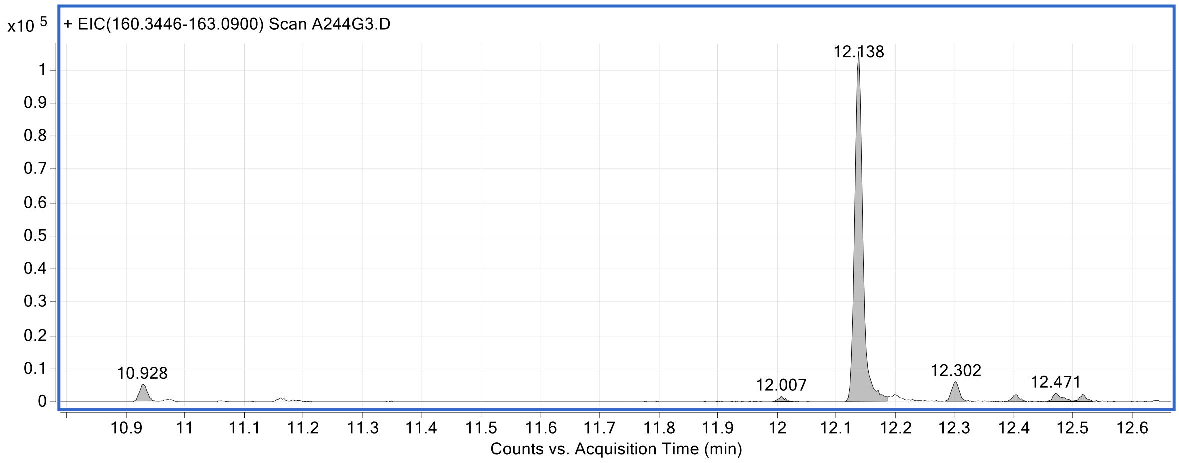

Figure S37: Extracted ion chromatogram (EIC) at m/z = 161 for A244G. 10.928 = germacrene D; 10.973 = α-cubebene; 12.238 = 10-*epi*-cubebol; 12.302 = cubebol; 12.471 = germacradien-4-ol; 12.500 = 1,10-di-*epi*-cubenol.

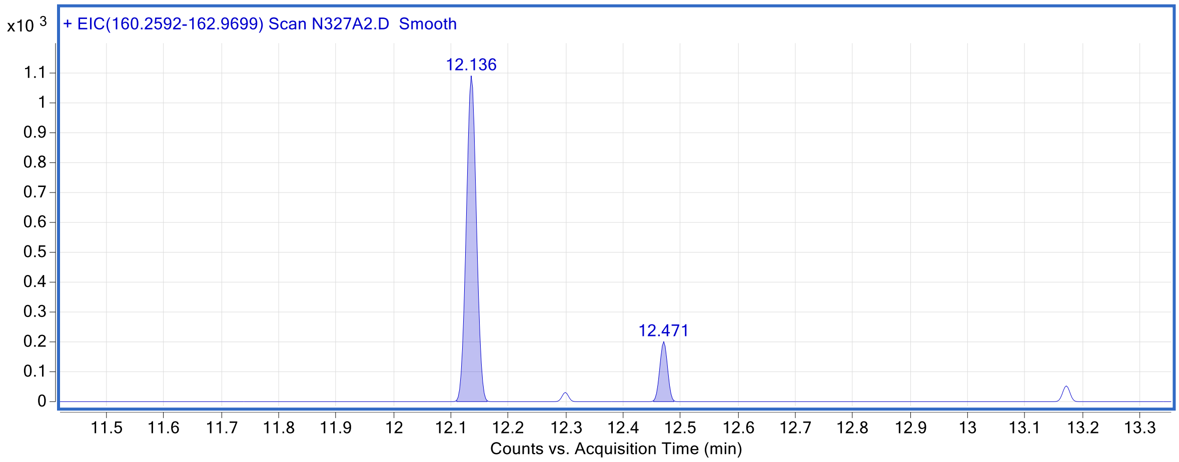

Figure S38: Extracted ion chromatogram (EIC) at m/z = 161 for N327A. 12.136 = 10-*epi*-cubebol; 12.471 = germacradien-4-ol.

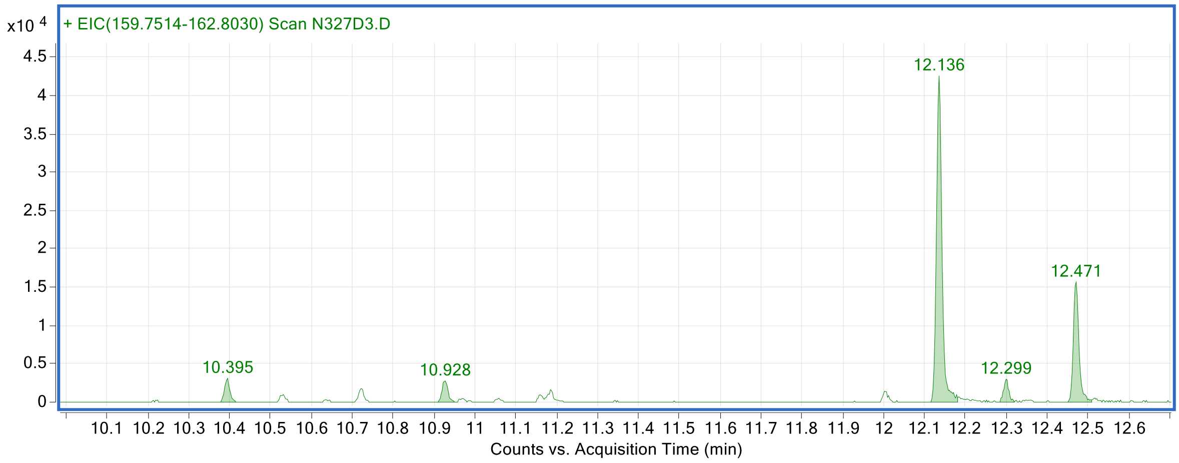

Figure S39: Extracted ion chromatogram (EIC) at m/z = 161 for N327D. 10.395 = germacrene D; 10.928 = cis-muurola-4,5-diene; 12.136 = 10-*epi*-cubebol; 12.299 = cubebol; 12.471 = germacradien-4-ol.

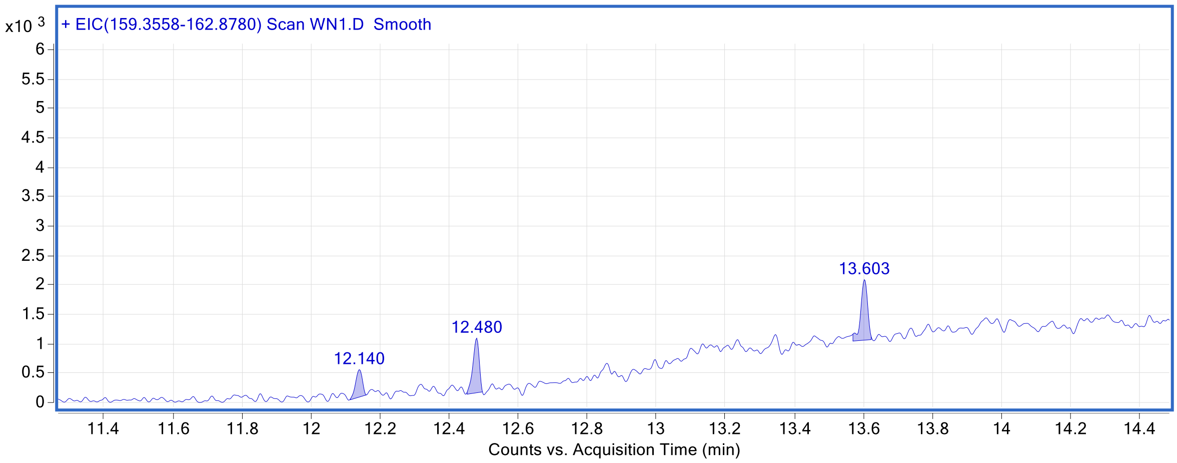

Figure S40: Extracted ion chromatogram (EIC) at m/z = 161 for F211W/N327D. 12.140 = 10-*epi*-cubebol; 12.480 = germacradien-4-ol; 13.603 = farnesol.

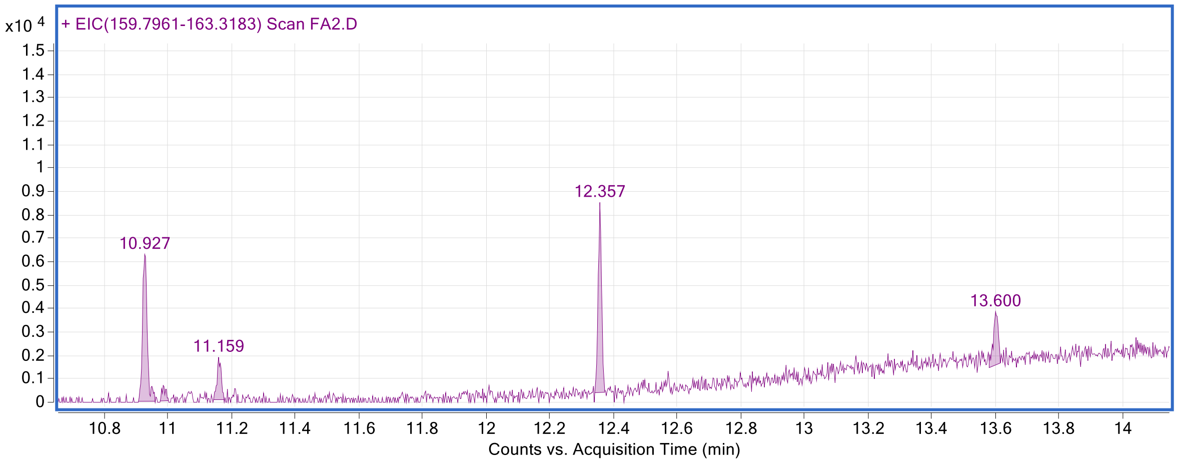

Figure S41: Extracted ion chromatogram (EIC) at m/z = 161 for F104A. 10.927 = germacrene D; 11.159 = γ-cadinene; 12.357 = nerolidol; 13.600 = farnesol.

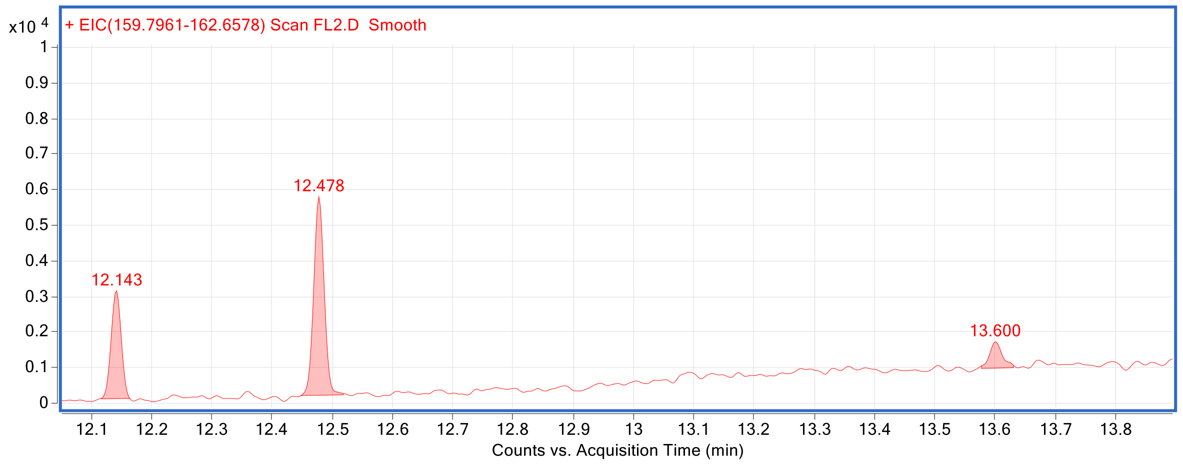

Figure S42: Extracted ion chromatogram (EIC) at m/z = 161 for F104L. 12.143 = 10-*epi*-cubebol; 12.478 = germacradien-4-ol; 13.600 = farnesol.

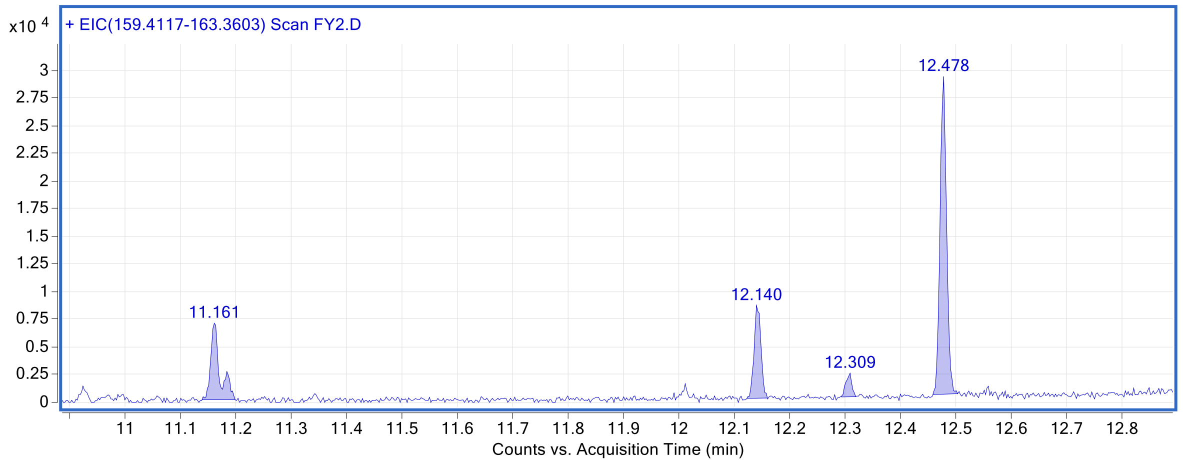

Figure S43: Extracted ion chromatogram (EIC) at m/z = 161 for F104Y. 11.161 = γ-cadinene; 12.140 = 10-*epi*-cubebol; 12.309 = cubebol; 12.478 = germacradien-4-ol.

References

[1] Schifrin, A.; Khatri, Y.; Kirsch, P.; Thiel, V.; Schulz, S.; Bernhardt, R.. A single terpene synthase is responsible for a wide variety of sesquiterpenes in Sorangium cellulosum Soce56. *Organic & Biomolecular Chemistry* **2016**, *14* (13), 3385–3393.

[2] Reddy, G. K.; Leferink, N. G. H.; Umemura, M.; Ahmed, S. T.; Breitling, R.; Scrutton, N. S.; Takano, E.. Exploring novel bacterial terpenes synthases. *PLOS ONE* **2020**, *15* (4), e0232220.

[3] Leferink, N. G. H.; Jervis, A. J.; Zebec, Z.; Toogood, H. S.; Hay, S.; Takano, E.; Scrutton, N. S.. A plug and play platform for the production of diverse monoterpene hydrocarbon scaffolds in Escherichia coli. *ChemistrySelect* **2016**, *1* (9), 1893–1896.

[4] Gorrec, F.. The MOPRHEUS protein crystallisation screen. *Journal of Applied Crystallography* **2009**, *42* (6), 1035–1042.

[5] Mccoy, A. J.; Grosse-Kunstleve, R. W.; Adams, P. D.; Winn, M. D.; Storoni, L. C.; Read, R. J.. Phaser crystallographic software. *Journal of Applied Crystallography* **2007**, *40* (4), 658–674.

[6] Emsley, P.; Lohkamp, B.; Scott, W. G.; Cowtan, K.. Features and development of Coot. *Acta Crystallographica Section D Biological Crystallography* **2010**, *66* (4), 486–501.

[7] Adams, P. D.; Afonine, P. V.; Bunkóczi, G.; Chen, V. B.; Davis, I. W.; Echols, N.; Headd, J. J.; Hung, L.-W.; Kapral, G. J.; Grosse-Kunstleve, R. W.; Mccoy, A. J.; Moriarty, N. W.; Oeffner, R.; Read, R. J.; Richardson, D. C.; Richardson, J. S.; Terwilliger, T. C.; Zwart, P. H.. Phenix - a comprehensive python-based system for macromolecular structure solution. *Acta Crystallographica Section D Biological Crystallography* **2010**, *66* (2), 213–221.

[8] Trott, O.; Olson, A. J.. AutoDock Vina: Improving the speed and accuracy of docking with a new scoring function, efficient optimization, and multithreading. *Journal of Computational Chemistry* **2009.**

[9] Guex,N.; Peitsch, M. C.. SWISS-MODEL and the Swiss-PdbViewer: An environment for comparative protein modelling. *Electrophoresis* **1997**, *18*, 2714-2723.

[10] Blank, P. N.; Barrow, G. H.; Christianson, D. W.. Crystal structure of F95Q epi-isozizaene synthase, an engineered sesquiterpene cyclase that generates biofuel precursors β- and γ-curcumene. *Journal of Structural Biology* **2019**, *207*, 218–224.

[11] Starks, C. M.; Back, K.; Chappell, J.; Noel, J. P.. Structural basis for cyclic terpene biosynthesis by tobacco 5-epi-aristolochene synthase. *Science* **1997**, *80.*

[12] Karuppiah, V.; Ranaghan, K. E.; Leferink, N. G. H.; Johannissen, L. O.; Shanmugam, M,; Ní Cheallaigh, A.; Bennett, N. J.; Kearsey, L. J.; Takano, E.; Gardiner, J. M.. Structural basis of catalysis in the bacterial monoterpene synthases linalool synthase and 1,8-cineole synthase. *ACS Catalysis* **2017**, *7*, 6268–6282.

[13] Baer, P.; Rabe, P.; Fischer, K.; Citron, C. A.; Klapschinski, T. A.; Groll, M.; Dickschat, J. S.. Induced-fit mechanism in class I terpene cyclases. *Angewandte Chemie - International Edition* **2014**, *53* (29), 7652–7656.

[14] Vedula, L. S.; Zhao, Y.; Coates, R. M.; Koyama, T.; Cane, D. E.; Christianson, D. W.. Exploring biosynthetic diversity with trichodiene synthase. *Archives of Biochemistry and Biophysics.* **2007**.

[15] Altschul, S. F.; Gish, W.; Miller, W.; Myers, E. W.; Lipman, D. J.. Basic local alignment search tool. *Journal of Molecular Biology* **1990**, *215,* 403-410.

[16] Wang, C.; Park, J.-E.; Choi, E.-S.; Kim S.-W.. Microbial platform for terpenoid production: Escherichia coli and yeast. *Biotechnology Journal* **2016,** *11*, 1291-1297.

[17] Ferraz, C. A.; Leferink, N. G. H,; Kosov, I.; Scrutton, N. S.. Isopentenol utilisation pathway for the production of linalool in Escherichia coli using an improved bacterial linalool/nerolidol synthase. *ChemBioChem.* **2021.**

[18] Baer, P.; Rabe, P.; Citron, C. A.; de Oliveira Mann, C. C.; Kaufmann, N.; Groll, M.; Dickschat, J. S.. Hedycaryol synthase in complex with nerolidol reveals terpene cyclase mechanism. *ChemBioChem* **2014**, *15* (2), 213-216.

[19] Babushok, V. I.; Linstrom, P. J.; Zenkevich, I. G.. Retention indices for frequently reported compounds of plant essential oils. *Journal of Physical and Chemical Reference Data* **2011**, *40*.
